# Preprints and Scholarly Communication: An Exploratory Qualitative Study of Adoption, Practices, Drivers and Barriers

**DOI:** 10.12688/f1000research.19619.2

**Published:** 2019-11-25

**Authors:** Andrea Chiarelli, Rob Johnson, Stephen Pinfield, Emma Richens

**Affiliations:** 1 Research Consulting Limited, Nottingham, NG7 2TU, UK; 2 Information School, University of Sheffield, Sheffield, S1 4DP, UK

**Keywords:** preprints, scholarly communication, peer-review, innovation diffusion theory

## Abstract

**Background**: Since 2013, there has been a dramatic increase in the number of preprint servers. Little is known about the position of researchers, funders, research performing organisations and other stakeholders with respect to this fast-paced landscape. In this article, we explore the perceived benefits and challenges of preprint posting, alongside issues including infrastructure and financial sustainability. We also discuss the definition of a ‘preprint’ in different communities, and the impact this has on uptake.

**Methods**: This study is based on 38 semi-structured interviews of key stakeholders, based on a purposive heterogeneous sampling approach and undertaken between October 2018 and January 2019. Interviewees were primarily drawn from biology, chemistry and psychology, where use of preprints is growing. Interviews were recorded, transcribed and subjected to thematic analysis to identify trends. Interview questions were designed based on Innovation Diffusion Theory, which was also used to interpret our results.

**Results**: Participants were conscious of the rising prominence of preprints and cited early and fast dissemination as their most appealing feature. Preprints were also considered to enable broader access to scientific literature and increased opportunities for informal commenting. The main concerns related to the lack of quality assurance and the ‘Ingelfinger rule’. We identified trust as an essential factor in preprint posting, and highlight the enabling role of Twitter in showcasing preprints.

**Conclusions**: The preprints landscape is evolving fast, and disciplinary communities are at different stages in the innovation diffusion process. The landscape is characterised by experimentation, which leads to the conclusion that a one-size-fits-all approach to preprints is not feasible. Cooperation and active engagement between the stakeholders involved will play an important role going forward. We share questions for the further development of the preprints landscape, with the most important being whether preprint posting will develop as a publisher- or researcher-centric practice.

## Introduction

### Background

The period since 2013 has seen a marked rise in the number of preprint servers set up for different communities in order to facilitate the rapid dissemination of pre-refereed research outputs. Tennant *et al*. (2018) list 18 servers launched between 2013 and 2018, variously set up by disciplinary communities, countries, research funders and publishers. One of the first in this new wave was the discipline-based server, bioRxiv – set up by the Cold Spring Harbor Laboratory in 2013 to cover the life sciences – which has been a focus of discussion and debate ( Abdill & Blekhman, 2019; Luther, 2017; Vale, 2015). However, there are a considerable number of other disciplinary servers, including several set up by the Center for Open Science, such as SocArXiv, engrXiv and PsyArXiv (all of which were launched in 2016), as well as platforms such as ESSOAr, set up by the American Geophysical Union in 2018. At the same time, national servers have been launched, including ChinaXiv (for China), IndiaRxiv (for India) and INA-Rxiv (Indonesia) ( Mallapaty, 2019). Funders of research have also set up platforms that enable the sharing of articles before peer-review, including, in 2016, Wellcome Open Research, for Wellcome-funded researchers. In addition, a number of journal publishers have added the dissemination of preprints to their workflows. The open access (OA) publisher, PeerJ, began offering preprint services in 2013, MDPI in 2016 and Cambridge University Press in 2019. Whilst the first of these has now closed its server, significantly it cites its reason for doing so as the change in the preprints landscape between 2013 and 2019: “the academic community is now well-served with other preprint venue options” ( PeerJ, 2019). A number of journals, primarily in biomedical sciences, have adopted a different model, and now deposit submissions from authors in bioRxiv on behalf of authors (where the author agrees to this). Journals practising this model in bioRxiv include *Proceedings of the National Academy of Sciences* (PNAS), titles published by PLOS and many published by Frontiers ( bioRxiv, n.d.). The *F1000Research* publishing platform has promoted a novel publication model involving preprints, in which immediate release of author submissions as preprints is followed by open peer review, with revised versions of a paper (alongside author responses to reviewer comments) published in the journal as they are made.

Of course, preprint servers as a venue of scholarly communication are not new. arXiv, the preprint server for physics, mathematics, computer science and related subjects, was set up as early as 1991 ( Larivière *et al*., 2014) – it is often regarded as an exemplar preprint server, and even the origin of the open access movement (Gajdacs, 2013). RePEc, housing ‘working papers’ in economics, was launched in 1997. There have also been unsuccessful attempts in the past to develop preprints services, for biology, as early as the 1960s ( Cobb, 2017) and again in the late 1990s ( Ginsparg, 2016), and chemistry, in the early 2000s ( Warr, 2003). *Nature Precedings,* an early publisher-driven preprints server was launched in 2007 and closed in 2012 (Nature Precedings, n.d.).

The move to set up servers since 2013 signals a new level of interest in preprints and a number of recent studies (e.g. Abdill & Blekhman, 2019; Balaji & Dhanamjaya, 2019; Luther, 2017; Tennant *et al*., 2018) have provided useful overviews of what Tennant *et al*. (2018, p. 5) call the “explosion of preprint platforms and services”. Significantly, this new wave of preprints has often come from disciplinary communities not previously associated with adoption of preprints. Biomedical disciplines served by bioRxiv, for example, have traditionally been associated with ‘Gold’ open access (publication in journals) rather than ‘Green’ OA (deposit of copies of papers in archives or repositories), and have typically not favoured dissemination of papers in pre-refereed form ( Martín-Martín *et al*., 2018; Wang *et al*., 2018). Usage of preprints in these new areas has varied across disciplines and servers but in some cases has been considerable, as evidenced by deposit rates. bioRxiv contained a total of 64,777 items on 12 November 2019. At that same time, ChemRxiv contained 2,892 items and PsyArXiv, 6,629 (although these two servers also contain items in addition to preprints).

The launch of these new preprint servers has led to discussion and debate, and some have suggested that preprints may become a disruptive force in scholarly communication ( Luther, 2017; Velterop, 2018). Green (2019) has argued for a digital transformation of publishing into a two-step process: articles would first be posted as preprints, and then invited to formal peer review only if they receive sufficient attention. He argues that this would not only represent a cost-effective model for OA and drive out predatory journals, but could also resolve the so-called ‘serials crisis’, under which growth in research budgets (which produce articles) consistently outstrips that of library budgets (which are used to purchase articles).

The case has been made for preprints in a number of disciplines, including biology ( Desjardins-Proulx *et al*., 2013; Fry *et al.,* 2019; Vale, 2015), medicine ( Lauer *et al*., 2015) and chemistry ( Carà *et al*., 2017). Some funders have signalled support for preprints being used in grant applications, including National Institutes of Health (NIH) and Chan Zuckerberg Foundation in the USA, and the Wellcome Trust in the UK.

However, sceptics have questioned the value of preprints and even suggested they may be dangerous – circulating versions of articles before they have been quality controlled by peer review may lead to unnecessary risk, particularly in disciplines like medicine ( Krumholz *et al.*, 2018; Leopold *et al.*, 2019; Sheldon, 2018).

This paper aims to explore the current and potential future role of preprints as a vehicle for scholarly communication by investigating current practices, drivers and barriers to their use. The overall objective of the study was to explore the place of preprints in the research lifecycle from the points of view of key actors, including:

- research funders;
- research performing organisations;
- preprint servers and service providers; and
- researchers (engaged and unengaged).

The topics in focus included usage of preprints, perceived benefits and challenges, policy positions, motivations and strategies. The research took the form of a set of 38 detailed interviews with representatives from these groups.

The study was funded by, and co-produced with, Knowledge Exchange (a group of national organisations from six European countries supporting research infrastructure), as part of their work on open-access policy and service development. It was, therefore, important that the research should not merely have a descriptive purpose but also a prescriptive one, involving setting out possible directions for future policy and action. The study is the first using empirical qualitative data focusing on the new wave of preprint servers set up since 2013, as such it aims to make a significant contribution to knowledge in this dynamic area.

### Literature review

Apart from recent discussion on the growth of preprint services ( Abdill & Blekhman, 2019; Balaji & Dhanamjaya, 2019; Tennant *et al*., 2018), consideration of preprints in the formal academic literature, as well as in the scientific press and other online venues (such as blogs and other social media commentary), has tended to concentrate on five main areas: firstly, defining preprints; secondly, their perceived benefits and challenges; thirdly, disciplinary differences; fourthly, policy developments; finally, the use and impact of preprints. We discuss these in turn in what follows. There are, however, still a relatively small number of empirical studies focusing on preprints, although the body of evidence is now growing rapidly. Nevertheless, much of the literature is still to be found in editorials and opinion pieces rather than data-driven research.

***Defining preprints***. Different definitions of preprints in the academic literature typically relate to a number of key components: (1) genre, (2) timing, (3) versioning, (4) accessibility, (5) responsibility and (6) value (see Table 1).

**Table 1.** Components of the definition of a preprint.

Component	1. Genre	2. Timing	3. Versioning	4. Accessibility	5. Responsibility	6. Value
Description	The type of output that a preprint is meant to be – part of the scientific literature	The position of a preprint in the knowledge production process – prior to formal publication	Preprints as different versions of an output at different stages of the outputs’ lifecycle – before peer review	The availability of preprints –preprints are openly available online	The individual(s) responsible for posting preprints – normally the author	The usefulness of preprints to readers – preprints are considered valuable
Exemplifying quotation	“A preprint is a complete scientific manuscript.” Berg *et al*. (2016, p. 899)	“A ‘preprint’ is typically a version of a research paper that is shared on an online platform prior to, or during, a formal peer review process.” (Tennant *et al*., 2018)	Preprints are made available “before, or in parallel to, submitting them to journals for traditional peer review.” Desjardins- Proulx *et al*.’s (2013, p. 1)	A preprint “can be viewed without charge on the Web.” (Berg *et al*., 2016, p. 899).	A preprint “is uploaded by the authors to a public server.” (Berg *et al*., 2016, p. 899).	“A preprint is a research output that has not completed a typical publication pipeline but is of value to the community and deserving of being easily discovered and accessed.” (Bourne *et al.*, 2017)
Other literature sources	(Balaji & Dhanamjaya, 2019; Bourne *et al*., 2017; Crossref, 2016; Luther, 2017; SHERPA/ RoMEO, n.d.)	(Carà *et al*., 2017; Johansson *et al*., 2018; Luther, 2017; Neylon *et al*., 2017; Rittman, 2018; Sarabipour *et al*., 2019)	(Bourne *et al*., 2017; Crossref, 2016)	(Johansson *et al*., 2018; Sarabipour *et al*., 2019)		

With regard to (1) genre, Berg *et al*. (2016, p. 899) state, “a preprint is a complete scientific manuscript”, and Bourne *et al.* (2017) observe, “typically, a preprint is a research article, editorial, review, etc.”. Whilst the latter widen the scope also to include, “a commentary, a report of negative results, a large data set and its description, and more” (p. 1), most of the discourse on preprints tends to assume they are conventional research papers and therefore follow the academic conventions of that ‘genre’ ( Kelly & Autry, 2013).

With regard to (2) timing, the key point made by most commentators is that a preprint is made available *before* formal publication, which Carà *et al*. (2017) describe as “prepublication”.

For (3) versioning, the relationship of a preprint to peer review is central. Desjardins-Proulx *et al*.’s (2013, p. 1) observation is typical in stating that preprints are made available, “before, or in parallel to, submitting them to journals for traditional peer review”. Suber (2012, p. 102) points out that this is not to “bypass peer review”, but that it applies to “works destined for peer review but not yet peer reviewed”. However, Bourne *et al*. (2017) controversially extend the definition to include “a paper that has been peer reviewed and…was rejected, but the authors are willing to make the content public”.

Accessibility (4) is crucial in definitions. A preprint is normally defined as being (or assumed to be) openly available online: it “can be viewed without charge on the Web” ( Berg *et al*., 2016, p. 899). The idea of openness is fundamental to discussions on preprints. The venue for distribution of preprints is often assumed to be a freely-accessible server of some kind, a point highlighted by Berg *et al*. (2016, p. 899), who include in their definition that a preprint “is uploaded by the authors to a public server”.

The above phrase, “by the authors” here is important and relates to (5) responsibility. Responsibility for distribution of preprints is traditionally assumed to be that of the author, a component of the definition that is often implicit in the verbs used to describe dissemination of preprints, such as, “sharing”, “posting” and “self-archiving”.

The final component of (6) value is summarised by Bourne *et al*. (2017): “a preprint is a research output that has not completed a typical publication pipeline but is of value to the community and deserving of being easily discovered and accessed”. To include that an output is “of value to the community” and is “deserving” of dissemination as part of the definition of what constitutes a preprint is interesting, since it includes a judgement of value in the definition. It would be difficult to demonstrate the value of each deposit as it is made. The idea of value is, however, one that is implicit in much of the discourse on preprints.

With ambiguities associated with each of these six definitional components, Neylon *et al*. (2017) are right that “no universal definition of preprints exists”. The label itself is ambiguous, composed as it is of ‘pre’ and ‘print’. The ‘pre’ of ‘preprint’ has sometimes been defined in relation to formal publication, with a preprint characterised as “prepublication”, leading to the controversial question of whether a preprint can itself be considered a ‘publication’ in its own right ( Larivière *et al*., 2014). More commonly, however, the ‘pre’ part of ‘preprint’ specifically refers to peer-review and is contrasted with ‘postprint’, a version produced *after* peer review. The conflation of peer review and publication in some discussions is a reflection of their close association in scholarly communication. It is interesting that the use of the terminology of ‘postprint’ in contradistinction to ‘preprint’, and with both termed generically as ‘eprints’, has declined in recent years. However, Tennant *et al*. (2018) have proposed its revival for reasons of clarity. Of course, the ‘print’ part of ‘preprint’ is largely anachronistic, but like terms such as ‘paper’ and ‘manuscript’, has continued to be used even in a digital environment.

***Perceived benefits and challenges of preprints***. Of the advantages of preprints discussed in the literature, perhaps the most prominent are the early and rapid dissemination of research results ( Khera, 2018). Using preprints has the potential to “accelerate” science, something that is particularly useful, for example, in combatting outbreaks of diseases ( Johansson *et al*., 2018). The formal scientific publication process is often seen as frustratingly slow, particularly in a context where final versions of articles may be little different from preprints ( Klein *et al*., 2016). Preprints allow authors to assert priority early – a preprint is date-stamped in a way widely recognised by many communities ( Ginsparg, 2016; Mallapaty, 2019; Tennant *et al*., 2019). Preventing researchers being ‘scooped’ is a major priority in many fast-moving disciplines, but applies, at least to some extent, in all areas of academic research, where novelty is prized. Early dissemination is seen by some as especially useful to a number of members of the scholarly community in particular, with early career researchers (ECRs) commonly identified as specific potential beneficiaries, as preprints can allow them to rapidly achieve “visibility” and demonstrate productivity in job and grant applications ( Desjardins-Proulx *et al*., 2013; Sarabipour *et al*., 2019; Tennant *et al*., 2019).

As well as being a fixed point in the scholarly discourse (date-stamped, etc.), another benefit of preprints emphasised in the literature is in the fact they are still subject to change. Authors can benefit from what Pinfield (2004) has called “informal peer review” of versions of their papers. Ginsparg calls this “crowdsourced peer review”, in contrast to “journal-mediated peer review”, and states, “authors benefit greatly from feedback from interested readers, contributing to improved versions of articles, which are then uploaded” ( Ginsparg, 2016, p. 5). There are, however, few empirical studies of such feedback and its value. There also appears to be little acknowledgement that the claim, when used to make the case for preprints in general terms, stands in tension to the one cited earlier that preprints often differ little from final published versions.

Other key advantages of preprints include wider and fairer distribution of research results, both within and beyond the academic community, something fundamental to many arguments for openness in general ( Ginsparg, 2016; Sarabipour *et al*., 2019). Access to preprints for machine-based crawling in order to facilitate text mining is also seen as an advantage by some commentators ( Chodacki *et al*., 2017). Furthermore, preprints, partly as a result of wider dissemination, can also increase numbers of citations of papers ( Davis & Fromerth, 2007) and create opportunities for collaborations ( Kleinert *et al*., 2018). Finally, preprint servers, their advocates argue, can sometimes usefully also house research outputs that might otherwise be ‘homeless’, including items that do not end up being published in peer-reviewed journals ( Bourne *et al*., 2017).

Perhaps the most prominent criticism of preprints relates to this last issue: the lack of quality assurance through peer review ( Sheldon, 2018). As well as a general concern about lowering quality standards, lack of quality control has been seen as potentially “dangerous” in, for example, areas such as medicine ( Krumholz *et al.*, 2018) as “reports that have not undergone formal peer review [organised by a journal] could be misleading” ( Lauer *et al*., 2015). Furthermore, uncertified science might be reported prematurely in the media and might even give rise to ‘fake news’ ( Sheldon, 2018). Some insist that, at the very least, the opportunity to disseminate knowledge rapidly without peer review may encourage academics to produce low-quality outputs on fashionable topics ( Teixeira da Silva, 2017). This issue, however, might be mitigated by the fact that authors sharing incorrect or low-quality research are at risk of reputational damage ( McGlynn, 2017), which could affect their career and future prospects.

Despite claims of the value of informal peer review enabled by preprints, some have pointed to the limited use of commenting and feedback features on preprint servers ( Sarabipour *et al*., 2019). Others have gone further and questioned the value of self-appointed reviewers, as opposed to those selected by journal editors (see the issue of “self-policing” highlighted by Harnad, 1998). Preprint posting, however, is not normally seen as a substitute for peer review, currently managed by journals, in filtering content ( Suber, 2012), a process that is commonly valued, even if recognised to be imperfect ( Lee *et al.*, 2013).

A number of authors report the concern that dissemination of a preprint may be considered ‘prior publication’, thereby jeopardising acceptance of the paper by a journal – the so-called ‘Ingelfinger rule’ ( Nallamothu & Hill, 2017). Whilst this convention has come under criticism and been withdrawn by many publishers, it still exists for some journals, e.g. in medicine and chemistry ( Lauer *et al*., 2015; Leopold *et al.*, 2019; Teixeira da Silva & Dobránszki, 2019).

It is noticeable that the literature on the pros and cons of preprints has, like many aspects of open science, given rise to robust discussion and debate. The paper by Krumholz *et al.* (2018) cited above is itself structured as a debate, with the first two authors making the case for preprints and the third expressing concerns. Sheldon’s (2018) opinion piece in *Nature* arguing that preprints could have a negative impact beyond the scientific community, was met with vociferous rebuttals in the letters pages of the journal the following month ( Fraser & Polka, 2018; Sarabipour, 2018; Tennant *et al.*, 2018). On social media, such as Twitter, there have also been vigorous exchanges (e.g. Twitter, 2019).

***Disciplinary differences***. Disciplinary differences have been a prominent feature of the literature on preprints. Neylon *et al*. (2017) in their seminal work conceptualising preprints usefully distinguish between the “state” and “standing” of preprints. “State” relates to, “external, objectively determinable, characteristics” of preprints; “standing” refers to the “position, status, or reputation” of preprints. Neylon *et al*. (2017) discuss in detail how preprints of similar states can have very different standings in different disciplinary communities, using the example of the contrast between physics and life sciences. For example, it is not universally agreed when an output should be citable (in the literature, funding proposals or promotion cases) or when it can be used to establish a claim of precedence. In disciplines where a preprint is not considered appropriate to establish precedence, it has also been suggested that making a preprint available may actually encourage research to be scooped by rival researchers who publish in a recognised journal before the preprint authors (the ‘flip side’ of the priority claim argument above) ( Kaiser, 2017).

It is commonly observed that physics has a well-established preprint tradition unlike many other Science, Technology and Medicine (STM) disciplines. Lauer *et al*. (2015, p. 2448) note, “biology…has trailed behind, whereas clinical research remains well behind.” Carà *et al*. (2017, p. 7924) characterise chemistry as being “late in embracing preprints”. Such language (“behind”, “late”) seems to represent an assumption that all disciplines will eventually come to use preprints, and that different disciplines are now simply at different points in the adoption process. Such a view has been disputed, however, with some arguing disciplinary differences in communication practices are likely to exist in the long term and therefore that preprints will not be adopted universally across disciplines ( Kling & McKim, 2000).

***Policy developments***. Of course, disciplinary practices do not operate in a vacuum. They are influenced, amongst other things, by the policy environment in which researchers work. Policies affecting researchers’ practices are developed by a number of groups: publishers, funders and institutions. Publisher policies are critical, with the Ingelfinger rule and deposit embargoes being examples of key policies that may have a negative impact on uptake in use of preprints. To this position of publishers resisting use of preprints, may now be added the contrasting recent development of some publishers embracing preprints, even setting up their own preprints services ( Callaway, 2013; Cambridge University Press, 2019). This development is not completely unprecedented, however, since it does build to some extent on well-established processes in areas like high-energy physics of integrating preprints into the journal submission process (as an example, some physics journals allow submission by simply pointing to an arXiv preprint).

Perhaps the most noticeable shift recently in terms of policy is that of funder policies. Some funders have now explicitly signalled support for use of preprints, including allowing citation of preprints in funding bids, and support their inclusion in cases for academic advancement ( Berg *et al*., 2016; Bourne *et al*., 2017). Very few funders, however, currently mandate use of preprints, although this has been proposed by preprints advocates ( Sever *et al*., 2019).

Institutional policy in this area shows some limited movement, with examples of institutions rethinking (usually rather cautiously) their approaches to criteria for career advancement in relation to the shifting scholarly communication environment, with some explicitly allowing submission of preprints ( ASAPbio, n.d.). It appears that, at present, many organisations still rely on metrics such as the journal impact factor when it comes to review, promotion and tenure of their staff ( McKiernan *et al*., 2019). Some have argued that initiatives such as Declaration on Research Assessment (DORA), with its emphasis on the quality of the output rather than venue of publication, promote use of preprints ( Polka, 2018), including acceptance of preprints are part of institutional researcher evaluation processes. Another interesting area of institutional policy is the positioning of the institutional repository (IR) in relation to preprints. IR policies and practices differ in this area, with many to date having focused on versions of outputs following peer review, although that is not a universal position ( Baughman *et al*., 2018).

***The use and impact of preprints***. A noticeable recent development in the literature has been publication of a number of empirical studies on the use and impact of preprints. These include Carneiro *et al.’s* (2019) study which compared the quality of reporting of findings in preprints from PubMed and bioRxiv against formally-published journal articles based on a number of criteria tested through a questionnaire. They found that the “quality of reporting in preprints in the life sciences is within a similar range as that of peer-reviewed articles, albeit slightly lower on average, supporting the idea that preprints should be considered valid scientific contributions”. Abdill & Blekhman (2019) analysed the growth in submissions to bioRxiv. Their work also shows a positive correlation between the use of papers on bioRxiv (measured by downloads) and the impact factor of the journal in which papers were subsequently published. Most recently, papers by Fraser *et al.* (2019) and Fu & Hughey (2019) have found evidence of a citation and altmetric score advantage for papers deposited in bioRxiv compared with those not made available as preprints. All of these studies were made available as preprints on bioRxiv in 2019 and, interestingly, all focus on bioRxiv, evidence of current interest in this growing service. They all notably present evidence of the positive impact of preprints. The papers usefully add to the growing empirical evidence base on preprints, augmenting studies such as Klein *et al*. (2016), on arXiv.

## Methods

### Analytical framework

As preprints and preprint servers are still innovative developments for most disciplines, it is important to gain an in-depth understanding of the perspectives of different stakeholders. In order to explore issues, such as varying motivations, differing behaviours, and conflicting perspectives, particularly in emerging areas, qualitative research methods are often deployed, since they are well-suited to such investigations. We chose to carry out detailed interviews of key actors in this space who could explain in depth their perceptions, attitudes and practices in relation to preprints. Participants were asked about their perspectives on preprints in general, but we intentionally recruited interviewees (where they had disciplinary affiliations) particularly from disciplines where preprint services are relatively new and rapidly growing. These were biology, chemistry and psychology, corresponding to the preprint servers bioRxiv, ChemRxiv and PsyArXiv. Focusing on these areas helped us to gauge the impact that preprints are having in areas where they are more innovative, and since many of our participants were able to speak more generally about preprints, we were able to draw comparisons with disciplines where preprints are established and which are better represented in the literature (e.g. physics, computer science, and economics).

As a way of framing our research design, we used innovation diffusion theory (IDT), a well-established theoretical framework for explaining the way innovations are adopted in different contexts ( Rogers, 2003). IDT has been tested and deployed widely and proved to be a robust explanatory model in a range of contexts, including OA ( Hampson, 2014; Jones *et al*., 2006; Pinfield & Middleton, 2016; Xia, 2012). It is designed to describe “the process by which an *innovation* is *communicated* through certain *channels* over *time* among members of a *social system*” ( Rogers, 2003) (original emphasis). An innovation is defined as “an idea, practice, or object that is perceived as new by an individual or other unit of adoption” ( Rogers, 2003). Preprints are both cultural innovations, as they aim to change practices in scholarly communication, and technological innovations, in terms of changes to infrastructures and processes. IDT offers ways in which these aspects of preprints as innovation can be understood, particularly in relation to two key issues: the “innovation decision process”, and the “rate of adoption”.

The innovation adoption decision process is seen as going through a number of consecutive steps:

1. **Knowledge**, when the decision-making unit is exposed to the innovation’s existence and gains an understanding of how it functions;
2. **Persuasion**, when the decision-making unit forms a favourable or unfavourable attitude towards the innovation;
3. **Decision**, when the decision-making unit engages in activities that lead to a choice to adopt or reject the innovation;
4. **Implementation**, when the decision-making unit puts an innovation into use; and
5. **Confirmation**, when the decision-making unit seeks reinforcement for an innovation-decision already made but may reverse the decision if exposed to conflicting messages about it. ( Rogers, 2003)

A particularly important concept to understand the success of innovations is their rate of adoption (see Table 2). This was used as the initial basis of the design of the interview questions. From the key factors associated with the rate of adoption, we selected a range of features that appear appropriate for the scope of the present investigation and that were suitable to discuss via interviews for the different stakeholder groups:

**Table 2.** Variables affecting the rate of adoption of an innovation (adapted from Rogers, 2003).

Variables determining the rate of adoption	Components of variables
Perceived attributes	Relative advantage
	Compatibility
	Complexity
	Trialability
	Observability
Nature of the social system	Norms
	Degree of network interconnectedness
Extent of change agents’ promotion efforts	Promotion efforts
Communication channels	Mass media
	Interpersonal
Type of innovation-decision	Optional
	Collective
	Authority

- **Perceived attributes**, are what stakeholders feel are the benefits arising from an innovation, in this case the introduction of preprints. Perceived attributes can be split into relative advantage, compatibility, complexity, trialability and observability.
- **Nature of the social system**, including “norms”, which are the established behaviour patterns for the members of a social system. They define a range of accepted behaviours and serve as a guide or standard for the behaviour of members of a social system. Norms tell individuals what behaviour they are expected to adopt and may be affected by the introduction of an innovation and by the actions of change agents within the social system. These relate to the level of interconnectedness or cohesiveness of the community.
- **Change agents’ promotion efforts**, which are the efforts made by individuals with influence in the system to promote the adoption of an innovation deemed desirable by a change agency (e.g. funders and institutions, service providers, publishers). Change agents often use opinion leaders in a social system as their lieutenants in diffusion activities.
- **Type of innovation decision,** which describes how the uptake of preprints is affected when individuals or communities support them, or authorities mandate their posting.

The topic of communication channels (additionally part of IDT theory on the rate of adoption) also arose organically from the discussions with our interviewees.

***Interview sampling and approach***. Interview questions were developed based on the factors outlined in Table 2 (see *Extended data* ( Chiarelli *et al*., 2019a) for more information). From an initial long list of possible questions, areas for investigation were prioritised based on the different stakeholder groups involved, and our review of the literature. We also incorporated questions associated to current policy-related issues, agreed in consultation with the Knowledge Exchange steering group, taking into account the innovation adoption process. This ensured that the approach taken was both theoretically robust and sufficiently grounded in practice to be useful in generating actionable insights. Interviews were conducted using a semi-structured approach – incorporating a ‘spine’ of common questions for all participants, and some questions specific to different actor groups – allowing room for the interviewer to pursue areas of interest arising from participant responses, including probing for greater clarity, where needed (Bryman, 2015).

The study adopted a heterogeneous purposive sampling approach, aiming to include a wide range of perspectives from actors in the area, selected in a “strategic way” in order to address the objectives of the study (Bryman, 2015). The sample was heterogeneous in a number of respects: firstly, it contained representatives of different roles in the scholarly communication system; secondly, it included participants from different countries (and therefore policy environments); thirdly, it comprised interviewees and with different views and levels of experience of using preprints. Participants comprised senior representatives from research funders, research-conducting organisations (universities and research institutes), preprint services, other related service providers (such as infrastructure providers), as well as researchers, both researchers demonstrably engaged with preprints (they had themselves posted a preprint) and non-engaged (there was no evidence of them having posted a preprint). In this study, we acknowledged the important role of academic publishers but chose not to engage them directly, apart from a sample running preprint services. This decision was made as the publishing community is already discussing preprints in a structured way, for example via the Committee on Publication Ethics (COPE).

Those participants with disciplinary associations (researchers and preprint service providers) came from the disciplines identified (biology, chemistry and psychology) but all participants were asked questions about preprints in general as well as their own community’s experiences. Participants were based in eight countries: Denmark, Finland, France, Germany, Netherlands, Switzerland, UK and USA, all apart from the USA and Switzerland being KE member countries. Participants were identified from the literature and from their associations with relevant services or organisations. Snowball sampling was also used as the research progressed and appeals for participation on social media were also shared (particularly to identify non-engaged researchers). Participants gave their informed consent and agreed to be named as participants in any reporting on the understanding that particular views or quotations reported would not be linked to them or their organisation and that the full text of transcripts would be kept confidential. The research approach adopted by the project was given ethical approval by the University of Sheffield. A full list of participants is available in Chiarelli *et al*. (2019b).

We undertook 38 semi-structured interviews, with participants distributed across the targeted stakeholder groups as illustrated in Table 3. The sample allowed the study to achieve the desired heterogeneity of actors and perspectives. Interviews took place between October 2018 and January 2019, and ranged from 32–75 minutes in length. They were conducted via GoToMeeting, recorded and fully transcribed using the intelligent verbatim method (including minor edits e.g. removing ‘fillers’ etc). Two interviews took the form of an email Q&A because of restrictions around the participants’ availability. The transcripts were then subjected to thematic analysis ( Braun & Clarke, 2006) which took place in several stages. Initially, members of the research team independently read a sample of transcripts from different stakeholder interviews, including some in common, and then discussed key topics arising from the transcripts. This formed the basis of the initial coding approach then undertaken by E.R. This was reviewed as analysis proceeded, with coding being checked and validated by A.C. and S.P. as it progressed and amended as necessary in light of their comments. The codes were then grouped into themes agreed by the team. These themes form the basis of the findings reported below.

**Table 3.** Participants by role and country (n=38). Participants are listed by Chiarelli *et al*. (2019b).

Stakeholder group	UK	Germany	France	Netherlands	Denmark	USA	Finland	Switzerland	Totals
**Research funders**	1	1	1	1	1	-	1	-	6
**Research performing organisations**	1	2	2	1	1	-	1	-	8
**Preprint server providers**	-	1	-	-	-	2	-	1	4
**Other service providers**	2	1	-	-	-	1	-	-	4
**Engaged researchers**	3	2	1	1	1	-	-	-	8
**Unengaged researchers**	4	2	1	1	-	-	-	-	8
**Totals**	11	9	5	4	3	3	2	1	38

***Limitations and constraints***. Like many kinds of qualitative research, this study was designed to be exploratory; in this case, to map out key aspects of the preprints space and suggest policy responses. Our conclusions are tentative. Many of our interviewees were selected because of their knowledge of the issues under investigation, and although our findings based on their views may be transferable to other contexts, they cannot be generalised without further testing, as with most qualitative research. There was a bias in our sampling towards participants aware of and engaged with preprints. Further research, using other methods, will be needed in future in order to generalise across communities as a whole, including non-engaged researchers. Furthermore, some stakeholder groups, such as publishers (who only have very limited representation in this study), and other groups (such as non-academic users of the research literature) could usefully be included in future studies. Our coding was undertaken using agreed protocols and involved a process of validation provided by three different members of the authorial team, but necessarily involves interpretation and judgement on the part of the researchers.

## Results

### Overview

The analysis of the data identified nine major themes arising from the interviews, which can be grouped into four thematic areas ( Table 4). These themes are used as the framework for presenting results and explored in more detail in what follows.

**Table 4.** Themes from the interviews.

Thematic area	Theme
Definitions and roles	Definitions of preprints
	Disciplines, cultures and practices
	Preprints’ position in the landscape
Potential benefits and challenges	Preprints as an asset (benefits)
	Preprints as a liability (challenges)
Infrastructure and sustainability	Infrastructure
	Policy
	Financial stability and business models
Future	Future of preprints

### Definitions and roles of preprints

***Definitions of preprints.*** In view of the ambiguities and disagreements in the literature around definitions of ‘preprints’, one key aim of the interviews was to ask our participants about their understanding of the term. Whilst all of the participants agreed with our broad definition that ‘a preprint is a research output made available in a form before it has been peer-reviewed and published’, there was considerable variation in the specifics of what that means. Some participants were themselves aware that the term was being used in different ways by different people and there were discrepancies (and in some cases confusion) about its precise meaning. One expressed dislike for the term, saying that it “presupposes…that you are in a print era”. However, other alternatives used by participants in this space, such as “manuscript” and “paper”, as has been observed, are equally anachronistic. There was evidence of participants struggling to find a clear language for the innovation being discussed.

Many saw a preprint as being in a form that was ready to be submitted to a journal, “at the point of submission” (Research performing organisation), as one participant put it. Referring to their own experience, one researcher stated:

*“the preprint…was only submitted or uploaded at a stage where it was essentially submission-ready for a journal.” (Engaged researcher)*

Other participants saw preprints as earlier versions of outputs made available in order to receive comments (e.g. working papers in economics). One participant acknowledged that a preprint was commonly thought of as the submitted version of an article but also discussed possible earlier versions being shared:

*“…in terms of quality, almost like [the] thing that will appear in the journal later on but if you consider a preprint like a working paper, you…definitely can see it as a step earlier in the whole research process in which there is still the possibility to enrich and improve the later formal publication on the same time.” (Research funder)*

Another participant referred to the benefit of this: “it gets feedback from the community” before “official peer review” (Unengaged researcher). A key point made by one participant, but implicit in the comments of many, was that that a preprint is a version of an output that has not been peer reviewed but that the author is “committing to get it peer reviewed” (Other service provider).

*“…the term itself includes the idea that you’re building it just towards something. That it’s only the preprint and then something will come later after from it.” (Research funder)*

Another author said of their own approach to posting preprints: “I always had the intent to publish this in a…proper journal subsequently” (Engaged researcher). A preprint is part of a “continuum” of different research stages, one participant argued, and authors should deposit all versions of their papers (and data) in a repository as part of contributing to that continuum. However, one participant did question this emphasis on a work flow with the preprint being provisional, since it appeared to devalue preprints:

*“…preprint means there’s something you know, there’s…a paradise afterwards, there’s a better life, and that it’s not a publication of its own.” (Other service provider)*

Either as a provisional version of a forthcoming publication or as a “publication” in its own right, a preprint was seen as part of a recognisable genre of scientific output, and which was planned to be part of the formal published literature, but was made available earlier than formal publication.

*“they’re not that radical, the concept is radical, but when you look at them, they look like articles.” (Other service provider)*

Several participants included post-refereed versions of articles within the definition (although some acknowledged these were not “pure” preprints), and others recognised that in reality many authors deposited post-refereed versions on preprint servers, usually to enhance the outputs’ accessibility. A small number of interviewees acknowledged that some papers posted as preprints might not end up being formally published, although this might raise questions about the value of the output:

*“If there’s nothing to follow the preprint then I would start to wonder what did happen. Why was the work dropped and left on this preprint level?” (Research funder)*

Some questioned whether a paper which did not end up being published could legitimately be considered a preprint, with one interviewee asserting, “it’s not a preprint; it’s just a manuscript” (Other service provider). That a preprint is basically a research paper was the assumption of the majority of participants, but one questioned even this:

*“You know, it’s basically anything, you know between a tweet…or a poster presentation and an actual paper…” (Engaged researcher)*

Many acknowledged there was uncertainty about preprints and interviewees were cautious in committing to a definition. In some cases, this related to disciplinary differences – something acknowledged by several interviewees. One interviewee discussed definitional differences between his own discipline, chemistry, and that of physics, mainly in terms of community acceptance. Another participant from the humanities stated:

*“I don’t think there’s any one perspective that scholars in the humanities have on preprints and I think that there’s some confusion about the terminology.” (Other service provider)*

***Disciplines, cultures and practices***. Disciplinary differences were evident not only in perceptions of what preprints are but also in terms of their acceptance. This was important throughout the interviews. There were firstly differing levels of awareness and, following this, adoption. Some interviewees recognized physics, mathematics and computer science to have well-established preprints practices with very high levels of awareness and adoption, but in other disciplines awareness was often still low:

*“I think in chemistry it’s small but growing and biology is being helped a lot by bioRxiv …there’s certainly some areas where there is still a kind of much less awareness. I would say really outside the math and physics [communities] the awareness is much lower of what preprints are about.” (Preprint server provider)*

One service provider summarized confusions (about definitions and processes) even applying to people trying to submit their work as preprints:

*“So there’s definitely a growing awareness but it’s still a minority. And we still find that there are some who are confused by the process and when they submit a preprint they don’t really understand. Despite everything we try and make them aware of, they don’t really understand the process.” (Preprint server provider)*

Furthermore, where there was evidence that awareness was rising, this did not necessarily result in uptake. Some unengaged researchers interviewed were aware what preprints were but had not been motivated to use them to date. For unengaged researchers, there were perceived practical barriers:

*“I am not entirely sure of the process, that’s the reason why I haven’t done it. I’m sure I could work it out but I’m not entirely sure which preprint server I would use, whether one would be better for my type of work than another…” (Unengaged researcher)*

However, there were signs this was beginning to change. One chemist described the situation in their discipline as moving from a position where there had previously been no use of preprints to where use was beginning to happen:

*“Almost all that is changing now and also from the chemistry part, which might be the related field, there was as far as I know ChemRxiv, which is like the main repository for chemistry data. It has been going on for one or two years maybe.” (Unengaged researcher)*

One participant described the process by which awareness of this new development diffuses through the community via informal channels and how they had become more personally aware of it during the course of doing a PhD:

*“I didn’t get any information on anything like, you know, I found out about it myself, you know, something as simple as that. Most of it’s just through word of mouth, like as you go through a PhD, as you’re talking to people, a lot of meetings, as you hear these terms come up more and more… It’s just accidental.” (Unengaged researcher)*

One participant representing a preprint server saw growing awareness and willingness to experiment in different subject communities:

*“And the momentum behind [name of the preprint server] – and I would not call it a success yet, I would call it momentum – but I think that momentum has given encouragement to other groups of scholars to investigate the possibility of developing a preprint platform for their own discipline. Whether that’s in earth sciences or anthropology or psychology, sociology and so on.” (Preprint server provider)*

This was confirmed by another service provider, who stated that before 2016, preprints “were not used much” in their discipline and many researchers “were unaware of what a preprint was”. But this was changing: “the popularity of preprints within the field…is rising” and this was partly attributable to the preprint server that the provider represented which had been a “driving force” for change (Preprint server provider).

The subject specificity of servers was seen as a natural way for preprints services to develop as it was seen as in line with the way researchers worked. However, even within disciplines, some referred to what they saw as significant differences between sub-disciplines, with some engaging in preprints and others not. Participants were agreed, however, that in disciplines where preprints were not established, although there might be some willingness to experiment, there was still a great deal of resistance:

*“I think lots of my colleagues use them just as much as I do but then I think there are some colleagues that would never post to a preprint.” (Engaged researcher)*

Finally, we note that adjacency between disciplines may play an important role. For example, disciplines close to those which posted preprints (e.g. those close to some areas of computer science or physics, which use arXiv consistently) may be more favourably disposed to the practice compared to those from other disciplines.

***Preprints’ position in the landscape***. Perceptions of the role of preprints in the scholarly communication landscape were partly derived from understandings of and sympathy for open science and open access developments. It was clear that those who were supporters of wider open science developments generally supported increased use of preprints.

We note that peer-reviewed journals are still seen as essential. As such, preprints were for most interviewees “part of a new ecosystem” (Research performing organisation), but not a radical departure from or replacement for selective journals, although their potential to prompt more fundamental change was highlighted by some (see below). The level of integration of preprints in processes associated with submission to a journal was generally seen as low, but there was some awareness of provisions, for example, where a preprint could be transferred to a journal’s submission system. For most disciplines, these were not seen as fundamentally important, however, in determining usage or take up decisions.

One researcher referred to ongoing “scepticism” in “many fields” (Engaged researcher). One of the key reasons for this was that the ‘standing’ of preprints in different fields was seen to be very different. This was reflected, for example, in different perceptions of the value of citing preprints. In some cases, researchers believed that citing preprints was not acceptable in either papers or grant proposals.

### Potential benefits and challenges of preprints

***Benefits.*** Participants highlighted a number of (potential) benefits and challenges of preprints which were seen to relate to particular practices around adoption or non-adoption of preprints. For the most part, these correspond to those identified in the literature, but it is useful to see how these are articulated and prioritized by our participants. Table 5 summarises the main benefits of preprints as highlighted in the interviews, comparing them with the literature. We have attempted to rank these by the prevalence of certain points across the entire dataset of interviews and have also classified them as to whether they create benefits at an individual or systemic level, that is whether they benefit individual researchers who practice them or the scholarly communication system as a whole. They are discussed in turn below.

**Table 5.** Potential benefits of preprints. Mentions across the entire dataset: ✔*** =over 20 mentions; ✔** =between 10 and 20 mentions; ✔* =fewer than 10 mentions. “Systemic” significance relates to those factors with system-wide impact e.g. the broad scholarly communication system or disciplinary community; “individual” relates to those factors primarily affecting individuals or small groups.

Benefit	Focus/ significance	Interviews	Literature
Early and rapid dissemination	Systemic	✔***	✔
Increased opportunities for feedback	Individual	✔***	✔
Preprint servers as an outlet for ‘homeless’ results	Systemic	✔**	✔
Advantages for early career researchers	Individual	✔**	✔
Preventing scooping	Individual	✔**	✔
Broader access to scientific research	Systemic	✔**	✔
Increased citation counts	Individual	✔*	✔
Preprints can support collaborations	Systemic	✔*	✔
Preprints in some formats (e.g. xml) and with open licences are easier to text and data mine	Systemic	✔*	✔
Much shorter time before research can be shared, so authors remain enthusiastic about it	Individual		✔
Preprints may reduce predatory publishing	Systemic		✔

Of the benefits highlighted by participants, early and rapid dissemination of research was the most frequent. As one engaged researcher succinctly put it:

*“The primary purpose of preprints is to communicate scientific knowledge as early as possible to as wide an audience as possible.” (Engaged researcher)*

Preprints were described by one researcher as enabling “science in real time” (Engaged researcher), particularly with reference to the lengthy period of peer review and publication that often applied to article publishing. One participant representing a preprint server described the peer review process as, “often long and tortuous” (Preprint server provider), and a university representative described it as a kind of “limbo” (Research performing organisation) in which the research was not being read or used.

Achieving rapid and wide dissemination could in turn “accelerate” the pace of research itself (Other service provider). This might mean being able to “see a result that somebody else did and I can start working on it” (Research funder), even though this type of behaviour was described as appropriate only in very fast-moving sub-disciplines. Research could also make progress thanks to the reduction of “redundant work”, which makes it more likely for researchers to identify “the next big research question” (Research performing organisation) more quickly.

The benefits discussed were mostly seen in terms of communicating with a particular disciplinary community, but some interviewees also mentioned reaching a wider audience, including policymakers or clinicians, in a timely way – research reflecting the latest thinking in an area has more “news value” (Research performing organisation). One engaged researcher also emphasized the benefit of making the latest research available in the case of an outbreak of disease.

Participants were conscious of the importance of dialogue and interchange as part of the research process and so valued the potential for preprints to create opportunity for feedback. Some participants conceived of this from the point of the researcher receiving comments on their paper in order to make corrections – a kind of “debugging” (Engaged researcher) – or other improvements:

*“…feedback from others to help you with your thinking and to improve your ideas.” (Research performing organisation)*

It was commented that some preprint servers facilitate this in various ways, by for example allowing authors to solicit feedback or providing commenting or annotation features. Many respondents saw this in wider systemic terms, rather than just personal: enabling community engagement and discussion – what one funder called “community-oriented discourse on research results” (Research funder). The language of ‘community’ was particularly strong amongst many participants here. Some provided stories of their experience of this, with one researcher commenting on a particular paper that,

*“the feedback from the community which we received through the preprint was at least as constructive and helpful as the official reviews from the journal.” (Engaged researcher)*

Many of the participants agreed that preprints servers could be a useful outlet for otherwise ‘homeless’ research outputs, even though this goes counter to the emphasis of many of preprints as early versions of outputs later formally published elsewhere. The most common sorts of outputs mentioned were null results and replication studies, which would not satisfy journal requirements of novelty or significance. However, participants also mentioned older or under-developed papers that had not been formally published but could easily be deposited on a preprint server and would be of value.

Such an approach could be particularly useful for early career researchers, who benefit in general from posting preprints in order achieve greater “visibility” relatively quickly. This could be particularly useful in the case of funding proposals or job applications in order to demonstrate productivity, although several participants were quick to emphasise that formal publications would be preferable. For all researchers, in fact, preprints were seen as possible evidence of productivity but no real substitute for formal publications.

The benefit of preventing scooping was also prominent – preprints were a way to establish priority:

*“If you’re an author the benefit of having a preprint in the public domain is it identifies the ideas as being yours, so it prevents you from being scooped because the ideas are down in time stamp against your name.” (Research performing organisation)*

However, a small number of participants observed that this might cut both ways, since in disciplines where preprints did not have the standing of a citable resource, making research available in this way might *cause* the researcher to be scooped: “if you put something online and it’s not yet published [I worry] that it would be stolen by competitors” (Unengaged researcher). The strength of the language (“stolen by competitors”) is telling of the intensely competitive culture in which many researchers work and is in marked contrast to the language of community noted above. However, other researchers were sceptical about whether scooping as a result of sharing preprints was likely to happen.

This was partly because a preprint is available so widely. Breadth of availability was certainly a benefit seen by many participants, primarily in reaching as many members of their own research community as possible. However, many participants also mentioned access by a wider readership, including the general public. Either way, the benefit of increased usage of papers was seen as particularly important. However, once again, the benefits were qualified, with greater value being placed on formal publications or accepted author manuscripts:

*“I think preprints are a very good kind of second option if you can’t access the published version and you can’t find the author’s accepted manuscript.” (Research performing organisation)*

A number of participants were confident making work available as a preprint would increase citations, with the paper being citable in preprint form if available on a preprint server. This applied particularly in fast-moving areas. Getting work out into the community at an early stage also, it was suggested, increased the opportunity to form new collaborations. This is in many respects the more optimistic side of the idea of competitors stealing ideas – the possibility that researchers may be encouraged to develop a collaboration as a result of seeing the developing work of peers.

***Challenges***. Participants also saw problems with preprints – summarised and compared with the literature in Table 6, with an indication of prevalence, and discussed in turn below. It is worth noting, however, that participants often had a nuanced view of the challenges, commonly stating potential disadvantages but then themselves qualifying their criticisms or citing possible solutions.

**Table 6.** Potential challenges of preprints. Mentions across the entire dataset: ✔*** =over 20 mentions; ✔** =between 10 and 20 mentions; ✔* =fewer than 10 mentions. “Systemic” significance relates to those factors with system-wide impact e.g. the broad scholarly communication system or disciplinary community; “individual” relates to those factors primarily affecting individuals or small groups.

Challenge	Focus/Significance	Interviews	Literature
Lack of quality assurance	Systemic	✔***	✔
Limited use of commenting/feedback features on the servers	Both	✔***	✔
Risk of the media reporting incorrect research	Systemic	✔***	✔
Possible harm in the case of sensitive areas	Systemic	✔***	✔
Questionable value of self-appointed reviewers	Both	✔***	✔
Information overload	Systemic	✔**	✔
The Ingelfinger rule – journals rejecting submissions if they have been posted as preprints	Individual	✔**	✔
Possible reputational damage to the depositor if the preprint is not good enough	Individual	✔*	✔
Possible ‘preprints wars’ in which the findings in one preprint are quickly attacked in another	Systemic		✔
There may be a rush to post low-quality research about popular topics	Systemic		✔

Of the problems, the lack of quality of assurance was most widely discussed by participants, with a set of related issues clustering around this theme. In some cases, there was a concern preprints could simply mean lower quality:

*“…so my worry would be with rapid publication by preprint that there would be an increase in the amount of poor quality science that’s available…” (Unengaged researcher)*

However, more commonly, there was the view that a lack of quality assurance meant greater uncertainty and that readers had a greater responsibility to approach preprints critically. Words such as “caution” and “sceptical” were often used. The filtering role of selective journals was valued by many participants. Peer review, for all of its faults (and participants were not slow to point out its possible failings), was still highly valued:

*“It’s really important that good reviewers have looked at the article and at the results section.” (Research funder)*

Peer review was thought of not just as a safety net but as a process which often improved the quality of a paper. Most of the supporters of preprints, did not therefore see them as an alternative to or replacement for peer-reviewed papers but as a complement to them. Whether or not they valued informal comments, participants commonly pointed out that the use of feedback functionality of preprint servers was still limited. One preprint service provider observed that only about 10% of preprints received comments.

Particular concerns were expressed around quality related to the media or members of the public latching onto unreliable findings, and on the harm a preprint containing errors could create in sensitive areas, especially associated with medicine or perhaps law. Several participants observed that people from non-academic contexts may not know the difference between a preprint and peer-reviewed article, and could therefore be more easily misled. Science journalists might potentially play a negative role. One participant said it was common for journalists to report findings from a peer-reviewed paper but “misinterpreting the results…and spreading the news without actually…understanding” the research (Engaged researcher). The risks associated with this were perceived to increase with preprints. Other participants acknowledged the responsibility of journalists, but were more optimistic, citing evidence of journalists using preprint servers responsibly and adding appropriate qualifiers to reports on preprints.

Several participants commented researchers themselves need to be aware of these problems and be cautious about how they published on controversial issues. Peer review did not mean that research papers were immune from such problems in any case.

There was a view expressed that basic screening provided by preprint servers, which was seen as very important by many participants, could address some of these concerns, but was still limited. There was consciousness that such basic checks themselves differed across different preprint servers and also that they would need strengthening in the case of servers dealing with medical outputs. One service provider working in this area suggested that preprint servers in the medical field might have to consider, among other things,

*“conflict of interest, financial disclosures, assurance that the work reported has been cleared by appropriate ethical review boards and assurance that data underpinning the article is available in an appropriate repository.” (Preprint server provider)*

This is a very interesting development which would involve preprint servers undertaking additional quality checks than just the current basic screening, potentially blurring the boundaries between them and peer-reviewed journals.

Participants were conscious of preprints creating what might be called a trust barrier. Whilst some of the determinants of trust for peer-reviewed papers might transfer to preprints (such as the overall shape of the paper, its authors, etc), some of the key determinants (the brand of the journal and its associated peer review processes) were missing. Participants were clear that preprints should be clearly marked as “not peer reviewed” and therefore treated with caution and handled responsibly. However, interviews also indicated possible contributors to trust that might apply to preprints, including: the preprint being widely discussed on social media, receiving comments online (e.g. on the preprint server), being cited, reported on by a magazine or newspaper, recommended by a colleague, and housed in a recognised preprint server.

Whilst there was enthusiasm amongst many participants for “community” based review and commentary on papers facilitated by preprint servers, there was some scepticism about the value of reviewers who “self-select themselves” (Unengaged researcher). People commenting may not understand the area or may use commenting to pursue personal agendas, it was suggested. In addition, an author might invite comments from people who could be expected to be positive about the work. The view was also expressed by some participants that the practice of commenting on preprints could cause difficulty with the formal peer review process, since people who had made public comments may be barred from undertaking blind peer review because of a conflict of interest.

There was concern also about information overload expressed by some participants, some of whom believed that preprints may inflate the number of papers being made available. However, other participants expressed scepticism of this, emphasising that the same number of papers were simply being made available earlier. There was also an acknowledgement that the filtering role played by selective journals was being removed from the process: some participants suggested that this could be at least partially solved with technology-based solutions, improving discoverability and filtering of content:

*“I think there is a lot of information out there but I think there’s also the potential to find technical solutions that will avoid the information overload.” (Preprint server provider)*

A number of participants pointed out that the solutions were better enabled by open, interoperable content, avoiding what one researcher called, “technical and legal restrictions put on by the publishers” (Engaged researcher).

Of these restrictions, the Ingelfinger rule was still the most commonly mentioned (although not by that name) by participants. However, there was considerable uncertainty and confusion amongst some participants (particularly researchers) about what authors could or could not do with regard to depositing preprints, and where they might find reliable information on what was permissible. Several researchers voiced such doubts:

*“I think there’s always the concern that people are worried that if I put something up there then it restricts where they can submit their paper.” (Engaged researcher)*
*“I have a duty to make sure that the work is peer reviewed in the best journals that we can get it into and if I rule certain journals out because I’ve submitted it as a preprint then I’ve kind of done a disservice to myself and those who I work with.” (Unengaged researcher)*

Such “fears” themselves acted as a considerable barrier to uptake. Interestingly, one engaged and one unengaged researcher did show an awareness of SHERPA RoMEO (a database of copyright and OA self-archiving policies of academic journals) as a source of information, but this was not common.

Apart from permissions barriers, there were also fears that reputational damage could arise from premature release of preprints. The consequence of this was that sharing a preprint would be delayed until the authors were confident in it to avoid the possibility of any reputational damage. One researcher commented:

*“I don’t think people in my field would just post off stuff that’s…terrible…because you’re still being judged on what’s going up there.” (Engaged researcher)*

In addition, where work was produced by a team of co-authors, gaining agreement from the team was itself an important quality barrier to overcome before disseminating the research in preprint form.

We note that some of the challenges arising from the posting of preprints were only perceived to be substantial in cases where researchers or re-users behave unprofessionally or unethically. However, this does not just apply to preprints, but also to other areas of scholarship and publishing. A key feature of many of the interviews was that participants were rarely able to cite empirical evidence of either benefits or challenges of preprints. At best, personal experience or anecdotes about the experience of colleagues was being cited. As one engaged researcher said:

*“I don’t have a lot of examples here, but certainly, you know, I hear anecdotes.” (Engaged researcher)*

Another said, “I have heard stories on Twitter” (Engaged researcher). It was apparent from many of the interviews that there is a need for further empirical work in this area in order to provide an evidence base for policy and practice.

### Infrastructure and sustainability

***Infrastructure.*** The view was commonly expressed by participants who commented on infrastructure that many technologies were already available to support use of preprints. Those mentioned by participants include repository and publishing solutions (open source, such as OSF Preprints, Eprints or DSpace and proprietary technology, such as Figshare), but also the broader scholarly communication infrastructure (e.g. indexing via Crossref). However, several infrastructural issues were emphasised as being important by participants, including technology considerations (such as interoperability, search and discovery), as well as process considerations (including licensing, versioning management, and digital preservation). Functionality and usability considerations for individual services were also mentioned, such as search and annotation of preprints.

Interoperability was regarded as a priority by many participants, with standards often being seen as key. Use of digital object identifiers (DOIs) for preprints (which could then be linked to later versions of the paper) was seen as a particular priority, but other standards such as ORCID, and service providers, such as Crossref, were also mentioned as being important.

Use of standards was seen as an important enabler of effective search and discovery of preprints. Discovery was seen as being achievable largely through network-level discovery services, such as Google Scholar, but interestingly, even greater emphasis was placed on social media, particularly Twitter. It was common for researchers to report finding out about preprints of interest from Twitter, either by following particular individuals or signing up to Twitter feeds set up and managed by preprint servers (including automated Twitter posting when preprints are released). Twitter was reported to be the main way several participants found out about preprints in their field. Several researchers also mentioned gaining feedback on their own preprints as a result of posting links to them on Twitter, with comments sometimes being posted on Twitter itself or other social media rather than the preprint server. The importance of Twitter was also emphasised by service providers, with one representative of a preprint server stating:

*“I would say that the momentum behind [name of the preprint server] owes a great deal to Twitter, and to Facebook, a bit less so. But nevertheless the effect is there. These are means of amplifying work once it has been posted.” (Preprint server provider)*

This is a significant finding. The fact that part of the infrastructure upon which preprint services are currently reliant includes generic discovery services, such as Google Scholar, and social media, particularly Twitter, needs to be taken into account in considering future developments of preprint services. Usage (or at least availability) of Twitter was assumed by participants to be widespread – a reasonable assumption in most Western countries, but not in others, particularly China.

Open licensing was also seen as an important infrastructural issue, which enabled interoperability and discovery. Preprint servers typically offer depositors Creative Commons (CC) licence options and there was some discussion in our interviews of the best one among these. Some authors were clearly confused about the options available to them when depositing their work. Some were aware of various requirements (including from funders) but were also wary of the possible consequences of signing particular licenses on a preprint when it comes to formal publication at a later stage.

Management of versioning was mentioned by a number of researchers although experience of this was mixed, with many authors not taking advantage of the facilities offered by preprint servers in terms of tracking versions. Preprint service providers were divided about whether these services allowed for withdrawal of items, but this was regarded as important by a number of participants in the case of misleading results or disputes between co-authors, for example.

Digital preservation, the final infrastructural issue discussed by participants at any length, was regarded as important in principle but often de-prioritised in practice because of its costly nature.

***Policy***. Participants identified key policy issues at different levels: publisher, funder and institutional. A cross-cutting issue applying to all policy levels discussed by many participants was, however, the value of preprints as reflected in and recognised by policy. Preprints were recognised to be valuable in providing early open access to research but their value in the scholarly record was commonly qualified by participants. Put simply, they were not considered as valuable as the author accepted manuscript (AAM) or the version of record (VoR), a view reinforced in the minds of many by the fact that many funder and government OA mandates did not have any requirements relating to preprints, but focused rather on AAMs and VoRs. This affected the extent to which different actors regarded preprints as a policy or operational priority. The concept of “standing” ( Neylon *et al*., 2017) is relevant here, with, as an example, standing relating to whether preprints are seen as an appropriate object for evaluation in exercises such as the UK’s Research Excellence Framework, about which there had apparently been uncertainty. One representative of a university stated:

*“they’re not acceptable for REF, so they’re not even part of the equation. So it’s the author’s accepted manuscript is the currency we deal with.” (Research performing organisation)*

The REF guidance was updated in January 2019 (whilst our interviews were still ongoing) and now states that preprints can be included in REF submissions, but expresses a preference for “final versions” of papers rather than preprints ( Research England, 2019). It is, however, also possible that institutions have developed local conventions for REF submission which in fact discourage submission of preprints, as can be inferred from this quotation.

The value of preprints was seen by many participants not so much in terms of their potential contribution to research evaluation exercises but rather in the extent to which they are a component of a more open research system. Whilst they were seen by some as a useful counterbalance to expensive OA publishing funded by APCs (article processing charges), mostly they were seen as important in terms of their contribution to the overall open science agenda:

*“…in terms of preprints, I’m more interested in a different problem, which is the problem of the opening up the whole research endeavour throughout the whole research process through the collection of data to the analysis of data to the curation of data through the writing research protocols. And then analysing the…the results and writing the software and all that stuff. Each of those things themselves should be considered a research output. The preprint is towards the end of that.” (Research performing organisation)*

Institutional-level policy was seen by many participants in this light. Preprints might be encouraged in a general way by institutional policy, but deposit of preprints would not normally be mandated. In fact, some participants reported that institutional policy was silent on the matter, and in practice preprints were not accepted as deposits to the IR.

Funders, similarly, often encouraged use of preprints and allowed them to be cited in grant applications, but they were not included as an acceptable form of an output which should be made available OA as part of a funder OA mandate. One preprint provider suggested funders might in future mandate use of preprints but pointed to only one small US funder who was currently doing so. There were other possible funders policies that could encourage change:

*“The other thing that funders really could do is to make very public the fact that they will allow a grantee to cite preprints in her progress reports and then her grant renewal application.” (Preprint server provider)*

Publishers’ policies were criticized by many participants as preventing authors from depositing preprints either before submission (the Ingelfinger rule) or after acceptance (contractual exclusions or embargoes). The environment was often seen as a confusing one for authors, something which was itself discouraging.

***Financial sustainability and business models***. Financial sustainability was a concern for many of the participants with one describing the financial sustainability of a number of preprint services as “fragile” (Research performing organisation), often being based on short-term project funding. One preprint service provider described the work they did in partnership with the Center for Open Science as based largely on the goodwill of “volunteers”. One participant commented that independent preprint servers have no business model associated with them other than grant funding. Although this was sometimes seen as a mark of immaturity, with experimentation in funding and sustainability models ongoing, some participants did point to problems in the funding of arXiv, a well-established preprint server, which had been through several periods of uncertainty with regard to its funding during its history.

Despite this, most participants seemed to favour a not-for-profit approach to preprint servers, some for reasons of sustainability, others emphasising the need for independence. Developments involving publishers becoming involved in delivering preprint services or in posting preprints on behalf of authors were, therefore, viewed with suspicion or even hostility by some participants. A number expressed concerns about the consolidation of services associated with academic workflows into the hands of a few commercial companies, including Elsevier ownership of SSRN, and Digital Science’s ownership of Figshare. This was seen as potentially jeopardising the independence of the relevant preprint services, even if they were currently operating in a standalone way. The language used by some participants to describe this development was in some cases strong, with one describing the sale of SSRN to Elsevier as “a huge betrayal of trust” (Engaged researcher) and another observing that this and similar developments have led to “power concentration” (Research performing organisation) in a small number of commercial providers. On the other hand, one representative from the preprint provider observed, “I don’t think that your average researcher thinks about that” (Preprint server provider); and it was indeed evident that many researchers amongst our participants did not show an awareness of such issues.

### The future of preprints

There was considerable uncertainty amongst participants about the future role of preprint servers. When questioned whether preprint servers could form a significant part of a system of scholarly communication which would be an alternative to or replace peer-reviewed journals, most were sceptical. There was, however, some discussion of the potential value of ‘overlay journals’ – where virtual journals are created from content held in preprint servers, having been peer-reviewed and selected after their circulation. Some suggested that automated filtering rather than human-based peer review might have a role to play in creating overlay services. There was some awareness of experimental work in the overlay area, but few were able to identify working examples.

It was clear from comments of participants that if preprints were to play a more significant role in scholarly communication, major improvements to the preprints infrastructure would be needed. This would include incorporation of preprints into scholarly and publisher workflows, provision for production of preprints in standards-based formats (e.g. XML) and greater consideration of preservation services. All of this would require major investment. However, even in a system where preprints did not replace existing channels of communication, such as journals, many of these developments were still considered necessary in order to make the preprint infrastructure more robust.

Whilst uptake of preprints was seen by many to be increasing, the role of policy in this area was uncertain. It was seen as particularly problematical for use of preprints to be mandated, as opposed to encouraged, by funders. There was a clear perception that preprints should be adopted by researchers who see the benefits themselves, rather than in response to a mandate:

*“There needs to be an intrinsic interest of the research community to communicate via preprints. I don’t think preprint posting can be enforced top-down or from anyone other than the research community and specifically the disciplinary communities themselves.” (Research funder)*

It is noteworthy that this view was expressed by a research funder.

## Discussion and conclusions

The findings of this research clearly relate to the innovation adoption decision process identified in Innovation Diffusion Theory, beginning with developing knowledge of innovation to confirmation of its adoption ( Table 7).

**Table 7.** Findings in relation to innovation adoption decision making factors from innovation diffusion theory.

Decision making step	Details – when the decision maker/ making unit…	Summary of current findings
1. Knowledge	…is exposed to the innovation’s existence and gains an understanding of how it functions	All interviewees chosen were already aware of preprints. However, we note that there is uncertainty around what ‘the rules of the game’ are: while people may be aware of preprints, the extent to which they are familiar with their value proposition varies.
2. Persuasion	…forms a favourable or unfavourable attitude towards the innovation	For some, the hypothetical advantages are clear, but they find it difficult to identify examples. Occasionally, there are doubts around whether posting a preprint may limit chances of publishing. Research institutions often do not have sufficient time and resources to promote and support preprints. In our sample, more interviewees showed a favourable attitude than an unfavourable one.
3. Decision	…engages in activities that lead to a choice to adopt or reject the innovation	In some cases, the Decision stage is strongly affected by the behaviour of peers, e.g. a co-author wishing to post a preprint. In other cases, the decision to post is supported by a belief in open scholarship and transparency. The failure to adopt the posting and/or reusing of preprints is often due to lack of uptake within a disciplinary community. In any event, ‘trialability’ is important before a decision is made.
4. Implementation	…puts an innovation into use	Experimentation was mentioned often in our interviews. It applies to preprint servers and overlay platforms in the first place, but also to some researchers who are trying to establish whether preprints can be beneficial to them – this is also related to the idea of trialability.
5. Confirmation	….seeks reinforcement for an innovation-decision already made but may reverse the decision if exposed to conflicting messages about it	Some interviewees reported that their or someone else’s preprints gained attention and feedback, particularly on Twitter. Feedback was sometimes received from important people within their disciplinary communities (e.g. researchers and editors), which may lead to an improved article, new connections or publication in prestigious journals.

It was evident that our participants were at different stages in the adoption process and this is a reflection of their peers’ and subject communities’ practices. Knowledge of preprints is rising but many are still not beyond the persuasion stage. Community norms remain crucial and have not shifted in many cases, therefore constraining individuals’ decisions. There was, nevertheless, some willingness to experiment, particularly amongst general OA supporters. There was some awareness of potential benefits becoming evident in practice but still at low levels. There was evidence of incomplete knowledge or misunderstandings amongst some researchers relating to preprints. The rate of adoption is influenced in our data by a number of factors highlighted in IDT ( Table 8).

**Table 8.** Findings in relation to the rate of innovation adoption from innovation diffusion theory.

Variable	Components	Summary of current findings
Perceived attributes	Relative advantage	The potential value of preprints is clear to most although the existence of a relative advantage for their use is not.
	Compatibility	The compatibility of preprints with existing practices and systems is perceived as variable and often unclear.
	Complexity	Perceived complexity of publisher policies and additional workload associated with deposit can be a disincentive.
	Trialability	Preprint servers can be a strong basis for experimentation.
	Observability	Some participants have observed the benefits of preprints based on colleagues’ experiences.
Communication channels	Mass media	The widespread use of Twitter is a noticeable characteristic which enables discoverability of preprints and growing awareness of preprint servers. It should be noted, however, that use of Twitter is not universal e.g. in China.
	Interpersonal	Personal recommendations are crucial in encouraging uptake, but were still often not present.
Nature of the social system	Norms	Preprints are dealt with differently based on whether people are early adopters of open science practices. In most other cases, preprints are considered as an important development, but scepticism still has to be overcome (e.g. with respect to practical advantages, funding streams and long-term preservation). Disciplines also play a significant role, as preprints are widespread in some research areas but only emerging in many others.
	Interconnectedness	Communities are important in shaping perceptions of the value of preprints. We found that two factors related to interconnectedness affect attitudes towards preprint posting: (i) the adjacency to disciplines where preprint posting is common (e.g. disciplines close to others within the scope of arXiv); and (ii) pressures and attitudes of close peers, particularly co- authors.
Extent of change agents’ promotion efforts		Promotion efforts are limited, and it is unclear whose role this should be. Stakeholders in the open science arena are promoting preprints within their circles and online. Some funders (e.g. EC, Wellcome) are making explicit efforts to promote preprints, but more significant and broader support (including from publishers) is likely to be required for higher uptake.
Type of innovation decision	Optional, collective or authority	Likely to continue to be optional for individuals influenced by their disciplinary community. Mandated uptake is seen as unlikely although encouragement from policy makers is likely to be strengthened.

It is worth mentioning that, in some cases, it is not clear whether researchers do not post preprints because there is no discipline-appropriate server for them, or there is no server because researchers in the field do not (want to) post them. The data shows that the willingness to be an early adopter of preprints may be related to sympathy with general open science and open access goals, as well as to the potential benefits researchers may see in their own use of preprints. Others remain sceptical, fearing that journals may reject their submissions, which is still the case in some areas ( Mallapaty, 2019), and questioning the value of circulation of pre-peer-reviewed outputs. The current environment for many disciplinary communities is therefore characterised currently by some experimentation, but also by uncertainty and fragmentation.

A key issue is trust. Trust is an essential feature of scholarly communication and was a recurring theme in the data, along with responsible posting and use of preprints. Nicholas *et al*. (2014) have shown that in the context of peer-reviewed journals, “researchers play down difficulties of establishing trustworthiness, not because there are none, but because they have well-developed methods of establishing trust”. However, preprints cut across those methods and create new ambiguities and uncertainties. The concern about lack of quality control and lack of quality indicators associated with preprints is fundamentally a matter of trust in a context where the “well-developed methods of establishing trust” are no longer present. Many of our participants were conscious that new norms were needed ( COPE Council, 2018) in this space and it was often the apparent absence of these that limited enthusiasm for preprints.

At a systemic level, the issue of sustainability emerged as critical from our work. Four models for delivering preprint services emerged from the data, each of which has different implications for sustainability and funding, an issue which was emphasised as being a concern by many participants:

1. Standalone preprint servers using in-house technologies e.g., arXiv;
2. Standalone preprint servers using third-party technologies e.g. bioRxiv using Highwire, ChemRxiv using Figshare infrastructure;
3. Publisher-supported preprints e.g. PeerJ Preprints (now closed), F1000; and
4. Publisher posting preprints to a preprint server e.g. PLOS partnership with bioRxiv.

Models 1 and 2 involve an ‘author-driven’ mode of posting preprints; Models 3 and 4 are a ‘publisher driven’ mode.

Model 1 is the ‘classic’ model of preprints, apparently assumed by most participants in their interviews. Model 2 is a version of 1 in which some infrastructure is outsourced to a third party, something which might help to enable sustainability by creating efficiencies through economies of scale (a common benefit of outsourcing). The infrastructure provided by the Center for Open Science is an example of such a benefit, with multiple preprint servers being run by the same organisation and on the same infrastructure, avoiding duplication, and therefore creating efficiency. Models 1 and 2 also include publisher-operated services such as Preprints.org and SSRN, which are owned by MDPI and Elsevier, respectively. These are not classified as publisher-supported (Models 3 and 4) but as standalone servers, because they still follow a paradigm of individual authors being responsible for posting their own preprints.

In this respect, Models 3 and 4 are different, since they involve the publisher rather than the author driving the preprint submission process, either through the publisher itself providing a preprints service as part of a journal submission workflow or by depositing on the author’s behalf. Models 3 and 4 move away from the traditional ‘author-driven’ preprints practices – where the author voluntarily deposits a paper as part of their own workflow, separately from submission to a journal – to a ‘publisher-driven’ preprints model, where the publisher to whom a manuscript has been submitted makes it available as a preprint. This is a fundamental shift which has major implications for the way the role of preprints is understood and the way preprints services are configured.

The future of preprints servers and their links with the overall scholarly communication process and infrastructure remain unclear. It is possible that the recent rise in preprint services might be reversed and that preprints go through a period of retrenchment, returning to serve the core areas traditionally associated with preprint use, such as high-energy physics. A possible alternative to such a ‘retrenchment scenario’ is what might be called the ‘patchiness scenario’, where different levels of adoption exist across different fields. Patchiness may be an ongoing situation, or may be a transition stage towards a possible ‘ubiquity scenario’. For preprints to become ubiquitous, of course, requires significant cultural and infrastructural change, some of which is indicated by the data presented here. This may partly depend on the integration between preprint services and other parts of the scholarly communication infrastructure and on related cultural norms. Closer integration may give rise to the possibility of more radical change in scholarly communication, creating opportunity for developments, such as overlay journals. Overlay journals have been discussed as thought experiments since at least the turn of the 21 ^st^ Century ( Smith, 2000; Smith, 1999) and there have been notable experiments in this area, and some ongoing services in specialised areas do exist, such as *Discrete Analysis* ( Ball, 2015). However, we are yet to see their widespread adoption, even though the potential remains.

Currently, the preprints landscape is rapidly changing, and disciplinary communities are at different stages in the innovation diffusion process. The high level of experimentation means that a one-size-fits-all approach to preprints is neither feasible or appropriate at present (if it ever will be). Something that will clearly play a role in the success of preprints (or lack thereof) is cooperation between the range of stakeholders involved. Though some of the issues we highlighted might appear independent of one another, we note that the majority of these affect multiple stakeholders at once.

**Figure 1.**
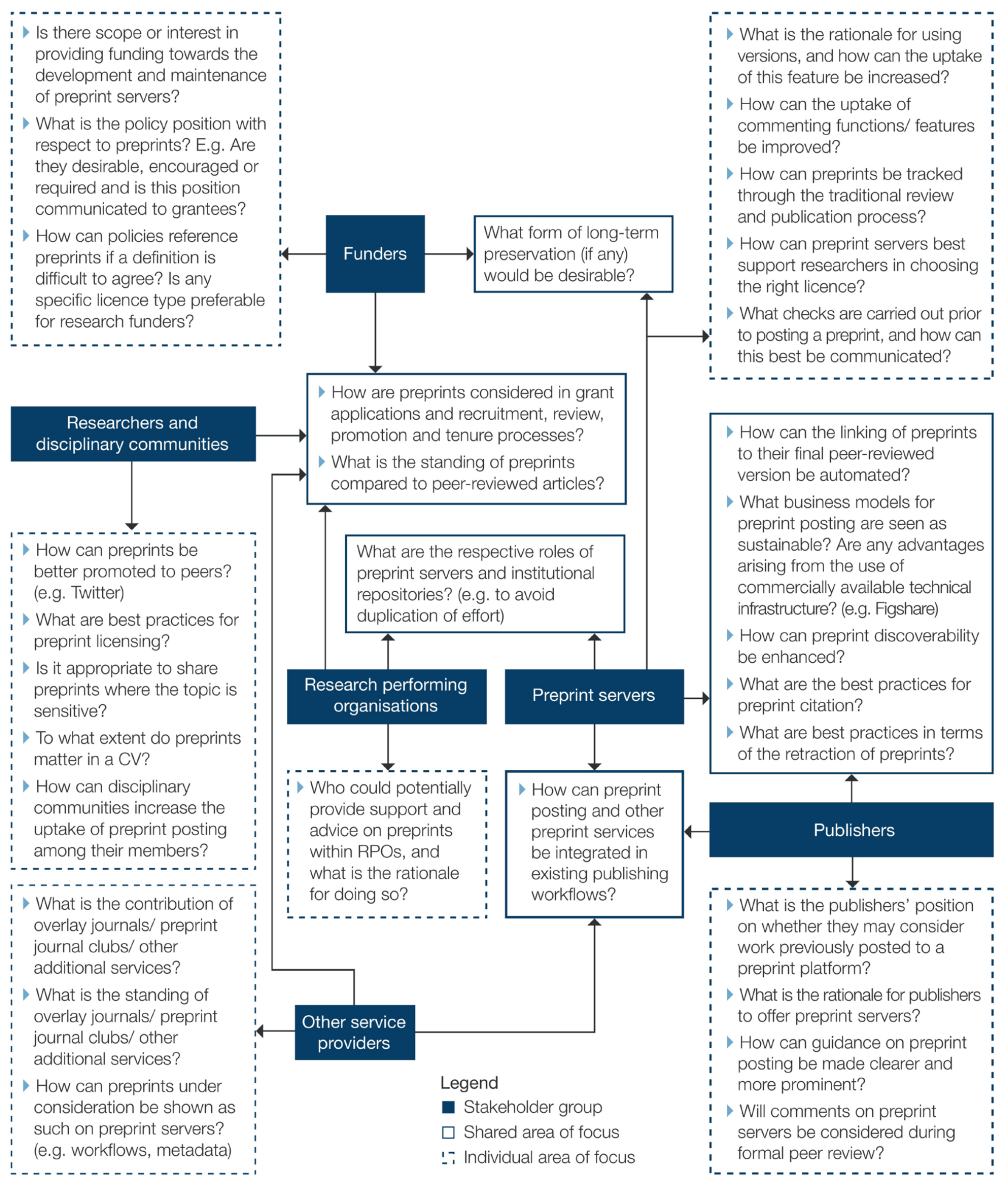
Questions to address in the preprints landscape ( Chiarelli *et al.*, 2019d).

Our findings have given rise to a number of key questions that we believe need to be addressed so that preprints can be supported sustainably in the future. Active engagement with these questions should lead to improved clarity and provide solid foundations for policy development and implementation. The questions and their relationships to the different actors involved are illustrated in Figure 1 (a tabular version of Figure 1 is available in Chiarelli *et al.* (2019c)). These include some key questions that need to be addressed by particular stakeholder groups, such as funders or preprint server providers. We have also added publishers as a separate group to this figure, taking account of issues that have arisen from the groups considered in this current study. However, there are also a large number of questions that need to be addressed through dialogue between different stakeholder groups. These are illustrated in the figure. One of the key challenges is to find channels for such dialogue to take place in order to develop solutions which are widely accepted.

This is in many respects an agenda for further research, discussion and policy design. We expect, following a rapid rise in preprints since about 2013, that many of these questions will come to be seen as increasingly important over the next five years. The urgency with which they are addressed, and the ways in which they are answered by the different stakeholders, will shape the role that preprints play in scholarly communications in the future.

## Data availability

### Underlying data

The authors confirm that, for approved reasons, some access restrictions apply to the data underlying the findings. Data underlying this study cannot be made publicly available in order to safeguard participant anonymity and that of their organisations. Ethical approval for the project was granted on the basis that only extracts of interviews would be shared (with appropriate anonymisation) as part of publications and other research outputs. In order to share data with other researchers, the participants must be contacted and consent to this data being released. In order to request data release, other researchers should contact the corresponding author or Chair of the University of Sheffield Information School Research Ethics Committee ( ischool_ethics@sheffield.ac.uk).

### Extended data

Zenodo: Interviewees and mapping of interview questions to areas of Innovation Diffusion Theory, http://doi.org/10.5281/zenodo.3538919 ( Chiarelli *et al*., 2019a).

Extended data.csv contains our interview questions, split by stakeholder group and mapped to innovation diffusion theory. List of interviewees.csv contains the names, roles and affiliations of interviewees.

Extended data are available under the terms of the Creative Commons Zero “No rights reserved” data waiver (CC0 1.0 Public domain dedication).
