# Supplementary material for: Preprints and Scholarly Communication: An Exploratory Qualitative Study of Adoption, Practices, Drivers and Barriers
Source: F1000Res. 2019 Nov 25;8:971. Originally published 2019 Jun 26. [Version 2] doi: 10.12688/f1000research.19619.2 (PMC6961415; doi:10.12688/f1000research.19619.2)
Supplement: Peer review history

# Peer review: Reviewer response for version 2

**DOI:** 10.5256/f1000research.23536.r58477

**Published:** 2020-02-04

**Authors:** Todd Carpenter

**Affiliations:** 1 National Information Standards Organization (NISO), Baltimore, MD, USA

This review of “Preprints and Scholarly Communication: Adoption, Practices, Drivers and Barriers,” covers the work by Chiarelli *et al.* to survey a group of 38 stakeholders in the preprint and scholarly community in a limited set of scientific domains, specifically, biology, chemistry and psychology.

**Methodology:**

 As a framework for assessing the adoption of Preprints, the Rogers model is a solid one, and the authors do a good job of crafting a survey around the five core elements of the basic conceptual model.

 One gap, and I feel it is an important one to be explicit about, particularly regarding Rogers’ conceptualization is the lack of focus on the discussion of motivations. Rogers defines this as the Knowledge-Attitude-Practice (KAP) Gap. To summarize, there is always a cost to make a change in any technical adoption process. The individual must be sufficiently motivated to make the change and thereby overcome the costs of making the change and adopting the new technology. With regards to motivations for adoption of preprints, this is a core element of the drivers of adoption, perhaps more so than the variables and findings outlined in Table 8 (which do follow Rogers’ approach). The potential ‘costs’ related to reputational damage, dissemination of erroneous results, intellectual property theft, or being blocked from formal publication. These costs change depending on the field and could correlate strongly with the willingness to use preprint systems.

 In table 2, the authors outline variables affecting the rate of adoption. Missing from this, based on KAP-Gap element of Rogers is the concept of the “conservative-ness” of the academic marketplace, where the risks and implications of failure can be significant. These risks increase in some academic domains, but remain regardless of the field. There is also a strong fixity and lack of receptivity of change in the academic social system which also inhibit adoption based on increased levels of ‘cost’ in the Rogers model.

 While this is not a critical flaw in the paper, focusing on this added element would draw out many of the themes that appear in a variety of the sections and give focus to the very real barriers that exist in the preprint marketplace. One can see that the authors skirt around this issue in some of their questioning and in their analysis. A stronger methodological framework would have highlighted these themes better.

 It seems that the interviews were intentionally selected to focus on particular domains and to include people with specific engagement with particular communities. While useful in a knowledge gathering process for a qualitative survey and the paper does a reasonable job justifying its selection rationale (page 8 & 9), the actual selection of participants seemed to be intentional, and this does add the concern about selection bias.

**References:**

 While I did not review the earlier draft, it appears that the literature review is reasonably balanced and covers a significant swath of the relevant literature, though to be fair this isn’t a review article and I don’t expect it to be comprehensive of every paper in this space.

**Results:**

 There is much within the results section to dig into intellectually and the feedback is useful qualitative reporting of the opinions of the subjects. Again, with the caveat that there is a potential for selection bias in these results, several additional details would improve the paper.

 Repeatedly, there references to approximations of the breadth of opinions expressed in the paper, such as page 10 “a small number of interviewees, or in table 5, where quantification of these amounts might be informative. I do understand that this is not a quantitative study and that including those figures might give that impression.

 There were inconsistencies or contradictions in a number of the responses, which are referenced in the reporting of the results, which is an indication of the variety of responses and perspectives covered in this survey. Some examples of these contradictions include the trust in preprints as a component of the publication workflow in which the manuscript would eventually be published, but yet preprint servers are also reported as a home for “orphaned” works that can’t find a ‘traditional’ publication home (page 13). The core value of preprints as a home for open access content delivery, but there is a rapidly growing acceptance of open access publication in the traditional space (page 13). That the readers of preprints should accept greater responsibility for assessing the quality of the papers, while also promoting their value as a source of information for the broader public, who likely are least well-positioned to assess the quality of a research output (page 14). The tension between the need for quality and trusted review of science and the desire to get results out the door as quickly as possible (page 14/15). This last tension is highlighted in the current environment by the rapid availability of manuscripts posted in preprint repositories on the coronavirus first identified in Wuhan, Hubei Province, China in the winter of 2020.

 I would have hoped for a fuller discussion and consideration of these results in the conclusion section of the paper. Again, I do not find this to be a fatal flaw of the paper. Perhaps the publication of these results will add to the urgency for a need to discuss these tensions of opinions related to preprints.

 The chicken-or-egg problem in the discussion section on whether the lack of repositories for a domain is limiting preprint deposit or the lack of demand is limiting creation of repositories. It seems in the current environment that much like there’s a journal for every paper, there is a repository for every preprint. This also seems out of place when the domains surveyed are ones in which specifically selected as having available repositories.

 Overall, the paper is well-written and well-reported. I recommend approval for indexing.

I confirm that I have read this submission and believe that I have an appropriate level of expertise to confirm that it is of an acceptable scientific standard.

# Peer review: Reviewer response for version 2

**DOI:** 10.5256/f1000research.23536.r58424

**Published:** 2020-01-13

**Authors:** Hamid Jamali

**Affiliations:** 1 School of Information Studies, Charles Sturt University, Wagga Wagga, NSW, Australia

This is an insightful and interesting article that reports the results of a qualitative study that includes interviews with key actors in the preprint domain in order to find out about the current and future role of the preprints. The study uses innovation diffusion theory as a framework. The paper is well-written and presents the results as it is the norm in social sciences. The literature review covers several aspects related to preprint that are relevant to the context of the research. The method has been described clearly and sufficiently. The findings are supported with evidence from the interviews (quotes) and they are discussed using the framework of the study (IDT). I found the comparison of the findings from the interviews with the literature and the questions for future studies (Figure 1) particularly useful.

 I also enjoyed reading the comments of previous reviewers and the authors’ response to those comments. I believe the methodology is fine and well-justified in the current version.

 I have no more corrections to suggest.

 As a minor point, RPO used in the figure hasn’t been mentioned in the paper and I had to find out what it stands for, although the phrase has been used in the paper.

I confirm that I have read this submission and believe that I have an appropriate level of expertise to confirm that it is of an acceptable scientific standard.

# Peer review: Reviewer response for version 2

**DOI:** 10.5256/f1000research.23536.r57003

**Published:** 2019-12-02

**Authors:** Jessica K. Polka, Naomi C. Penfold

**Affiliations:** 1 ASAPbio, San Francisco, CA, USA

The authors' additions greatly enrich and clarify this version of the manuscript. While our queries were addressed, the additional description makes it clear that we lack the expertise to evaluate the methodology. We therefore recommend that a third reviewer with appropriate disciplinary expertise take a look at the revised manuscript.

We confirm that we have read this submission and believe that we have an appropriate level of expertise to confirm that it is of an acceptable scientific standard, however we have significant reservations, as outlined above.

# Peer review: Reviewer response for version 2

**DOI:** 10.5256/f1000research.23536.r57004

**Published:** 2019-12-02

**Authors:** Sarvenaz Sarabipour, Humberto Debat

**Affiliations:** 1 Institute for Computational Medicine, Department of Biomedical Engineering, Johns Hopkins University, Baltimore, MD, USA; 2 Center of Agronomic Research, National Institute of Agricultural Technology (IPAVE-CIAP-INTA), Córdoba, Argentina

The authors have adequately responded to our comments.

We confirm that we have read this submission and believe that we have an appropriate level of expertise to confirm that it is of an acceptable scientific standard.

# Peer review: Reviewer response for version 1

**DOI:** 10.5256/f1000research.21512.r50950

**Published:** 2019-08-05

**Authors:** Jessica K. Polka, Naomi C. Penfold

**Affiliations:** 1 ASAPbio, San Francisco, CA, USA

In “Preprints and Scholarly Communication: Adoption, Practices, Drivers and Barriers,” Chiarelli and colleagues present a literature review, commentary, and a summary of attitudes and perceptions towards preprints found by interviewing 38 stakeholders. The interviews convey many commonly-held perceptions about the benefits and disadvantages of preprints, and the authors suggest questions for further exploration and testing. With revisions (clarifying the origin of claims, contextualizing the responses from study subjects, and correcting factual information in the text) this paper will become a very useful addition to the literature. 

**Major comments**

- Design and Subjects:
   
  - We encourage authors to rely on a more robust review of published literature (including the grey literature) to complement their interviews, as the published literature contains useful context for comments which are obfuscated by the process of anonymous reporting (For example, the sentence, “One preprint service provider observed that only 10% of preprints received comments.” would be more useful to readers if accompanied by a reference with attribution: https://asapbio.org/biorxiv)
  - Since this work may be of interest to a wide range of readers, we recommend including more detail regarding the chosen experimental design. 
    - Please provide justification for the experimental design, for example, why were interviews used instead of a survey? 
    - What are the methodological considerations that would be helpful for readers outside of qualitative research to understand: limitations, constraints, advantages over more quantitative methods? 
    - Please define semi-structured interviews — how were researchers encouraged to expand on thoughts? 
    - Please define “purposive heterogeneous sampling approach.”
    - Please clarify why the sample size is 38.
  - Please provide more information about the subjects to help readers assess relevance:
    - What is their geographical context?
    - How knowledgeable were subjects, and do they bring bias for or against open science into their statements? 
    - How did interviewees arrive at their opinions? How much close experience do they have with preprints? Were participants asked to answer only within their own expertise? 
    - There are quoted statements that reflect inaccurate understandings and are not always qualified by the author: for example, “relating to whether preprints are seen as an appropriate object for evaluation in exercises such as the UK’s Research Excellence Framework, about which there remains ambiguity. One representative of a university stated: “they’re not acceptable for REF, so they’re not even part of the equation. So it’s the author’s accepted manuscript is the currency we deal with.” (Research performing organisation)”  This is inaccurate and misleading (see https://www.ref.ac.uk/publications/guidance-on-submissions-201901/) but there is no further commentary from the authors. Please add clarification that the interviewee responses are not necessarily factual. (It would be interesting to compare interviewee opinions with known facts to understand which topics are well-understood.)

 

- Clarity of presentation:
   
  - Please provide more quantitative context to the opinions expressed. For example, for phrases such as “Some researchers” it would be useful to be more specific. Please also be more quantitative in assessment of the themes — it is useful to know if a sentiment was expressed by a majority or minority, as shown clearly in tables of benefits and challenges. Please provide aggregate data of thematic analyses, not just quotes, and use specific numbers and proportionality (Two of Five researchers mentioned X as a benefit).
  - The paper combines elements of a literature review, white paper/policy piece, and a qualitative study. It would be helpful to clarify the origin of ideas by separating the work clearly into Results and Discussion sections. (As one example, under the heading “Financial sustainability and business models,” it is unclear whether the proposed taxonomy was expressed by interviewees or generated by the authors. Similarly, it is unclear whether use of the term “seminal” comes from interviewees or authors.)
  - Readability:
    - Please use active voice and concise language to improve readability.
    - Bring interview questions and list of participants into this report — it is hard to interpret findings while having to continuously refer back to other sources, e.g. the 2019b interview structure paper.
- Claims:
   
  - The abstract sets the reader up to expect a review of preprint servers that have emerged since 2013. However, the methods section discloses that coverage of the study is limited to biology, chemistry, and psychology. It would be helpful to readers to state this up front. It is also unclear how this scope was applied in the selection of study subjects. It is difficult to determine if the scope influenced the selection of participants as listed in https://zenodo.org/record/2654832#.XTeUB5NKjs0 – the questions listed at https://zenodo.org/record/3240426 do not seem specific to these three disciplines and the quoted answers in this report suggest interviewees’ responses were not limited to these three disciplines. Please clarify how the scope of the interviews was constrained to the stated subjects, or if not, please revise the stated scope to more accurately reflect the scope employed.
  - The abstract does not reflect the content. The interview responses are summarised in general terms, not related to defined scope or the newer servers. A substantial proportion of this article is review material: either in the explicit literature review section, or as author-contributed content interspersed with summaries of interviewee responses. For example, conclusions listed in the abstract appear to be drawn from the literature review.
  - “Our study is the first using empirical data to understand the new wave of preprint servers” — this is not supported by the study content: no results are specifically related to the newer servers identified in the introduction; quoted excerpts indicate that interviewees responded in general about all servers and disciplines. Furthermore, prior surveys and bibliometric analyses have looked at a subset of these servers, either individually or in groups (eg https://osf.io/5fk6c/). ^1^

**Minor comments**

 We recommend making the following amendments to improve the clarity and accuracy of the report.

 In the Introduction and literature review: 

- The summary of the longer history of preprint efforts across disciplines, would benefit from incorporating and citing ‘The Brief Prehistory of Preprints” (Cobb, 2017) ^2^
- Zuckerberg Foundation should be corrected to Chan Zuckerberg Initiative.
- Discussion of new servers would be improved with a note as to their current size, and with awareness that not all new ‘preprint’ servers are exclusively for preprint content (many COS servers include postprints that are not possible to easily distinguish from preprint versions).
- “This paper aims to explore…by investigating current practices, drivers and barriers to [preprint] use.” The interviews indicate perceptions and attitudes but do not reveal actual behaviours or practices. Please clarify how this study examines current practices.

Figures and tables:

- Fig 1: To increase accessibility, please provide as raw text with headings or tags to indicate which stakeholder group you believe is most relevant

Literature review:

- The authors focus on four main issues — how were these arrived at? Was the literature review systematic in any way? 
- Given the emergent phase of preprint infrastructure in biology, chemistry and psychology, and as the authors note that empirical data is lacking, it may be equally valuable to include more grey literature (blogposts, webpages) in this review, given that several of the cited articles are opinion pieces, albeit as editorials or peer-reviewed review articles. Of note, we find personal blogs are a useful source of stories that detail benefits and drawbacks of preprints from personal experiences.
- "Dis-benefits:" is not a common term, perhaps use “drawbacks,” “concerns,”, “disadvantages” or “potential risks”.
- When discussing the lack of quality assurance, the authors seem to make an implicit assumption here that journal-led peer review assures quality. It would be fair to contextualise this section with some literature on peer review to understand variety of peer review outcomes on quality of paper and validity of findings.

Results:

- In the discussion of preprints in the medical field, please note that the concerns raised are being addressed by medRxiv. https://www.medrxiv.org/
- In the table of business models:
   
  - Note that bioRxiv uses 3rd party technology, Highwire.
  - “Publisher driven” models are not new; PeerJ is older than bioRxiv, Nature had Nature Preceedings in the early 2000s, and bioRxiv has had J2B preprints since its inception.
  - How does the outsourcing of infrastructure to a third party enable sustainability? Arguably, depending on the third party, this leaves the service vulnerable to changes in service entirely outside its control.
  - Individual authors can still submit to PeerJ.
  - Most publisher-mediated preprint submissions are voluntary for authors (a notable exception being F1000).
  - Regarding the statement of subject preferences: we have conducted a survey on these preferences that may provide additional context: https://asapbio.org/asapbio-funders-workshop 

**The following suggestions may also help improve the paper but are not essential.**

 In the Introduction and literature review:

- Open Research platforms could be discussed in context of F1000Research, since this is the model they are derived from (in collaboration with F1000).
- Please clarify the age of PeerJ and Preprints.org in comparison with bioRxiv.
- PLOS is not the only journal that deposits preprints to biorxiv using the J2B mechanism — several do, please refer to the list of journals offering direct transfers to bioRxiv ( https://www.biorxiv.org/about-biorxiv).
- Regarding trust in preprints, it would be relevant to mention or cite the current effort by COS to better understand indicators of trust in preprints: https://cos.io/about/news/center-open-science-receives-grant-alfred-p-sloan-foundation-study-trust-and-credibility-preprints/.

Figures and tables:

- Fig 1 introduces some interesting questions, however many of these are related to topics that are brought up for the first time in this figure.
- Table 6: The concern of reputational damage is indeed in the literature; please see point 5 here: https://smallpondscience.com/2017/07/24/whats-up-with-preprints/ 

Literature:

- “However, Bourne et al. (2017) controversially extend the definition to include “a paper that has been peer reviewed and…was rejected, but the authors are willing to make the content public”.” — Please provide reasoning or citation as to why this is controversial.
- Accessibility — note that preprints are not necessarily open access, where they are not provided with permissive licensing, and this is certainly the case for bioRxiv. ^3^
- One potential benefit to ECRs that is not mentioned here is being able to demonstrate productivity to funders and hirers. This would be useful to append to the current paragraph, given it is raised in the interview questions.
- “There also appears to be little acknowledgement that the claim stands in tension to the one cited earlier that preprints often differ little from final published versions.” — There is only tension if both claims concern all preprints. It is possible for there to be preprints that improve through a lot of feedback as well as preprints that undergo no/very little change.
- Please use unshared or unpublished instead of “homeless”.
- We recommend avoiding the term “policy stack,” as there is no dependent relationship among these different groups/stakeholders.

Methods:

- This section ignores the efforts of researchers as change agents.

Results:

- Please providence evidence for this statement: “we note that adjacency between disciplines appears to be playing an important role.” 
- “Many of the participants agreed that preprints servers could be a useful outlet for otherwise ‘homeless’ research outputs, even though this goes counter to the emphasis of many of preprints as early versions of outputs later formally published elsewhere.” — this attitude may reflect a lack of publication venues for such content, not a lack of desire to publish these other outputs in a peer-reviewed destination.

We confirm that we have read this submission and believe that we have an appropriate level of expertise to confirm that it is of an acceptable scientific standard, however we have significant reservations, as outlined above.

## References

[1] : Quantifying the growth of preprint services hosted by the Center for Open Science.2019; 10.31219/osf.io/5fk6c 10.31219/osf.io/5fk6c

[2] : The prehistory of biology preprints: A forgotten experiment from the 1960s. *PLoS Biol*.2017;15(11) : 10.1371/journal.pbio.2003995 e2003995 10.1371/journal.pbio.2003995 29145518PMC5690419

[3] : Biologists debate how to license preprints. *Nature*.2017; 10.1038/nature.2017.22161 10.1038/nature.2017.22161

# Response

**Published:** 2019-11-14

**Authors:** Andrea Chiarelli

**Affiliations:** Research Consulting, UK

*In “Preprints and Scholarly Communication: Adoption, Practices, Drivers and Barriers,” Chiarelli and colleagues present a literature review, commentary, and a summary of attitudes and perceptions towards preprints found by interviewing 38 stakeholders. The interviews convey many commonly-held perceptions about the benefits and disadvantages of preprints, and the authors suggest questions for further exploration and testing. With revisions (clarifying the origin of claims, contextualizing the responses from study subjects, and correcting factual information in the text) this paper will become a very useful addition to the literature.*

**Authors’ response:**

We are grateful for the reviewers’ summary of and comments on our article, and are pleased that they consider that “with revisions…this paper will become a very useful addition to the literature.” The above summary makes a number of general remarks about revisions that should be made which we address below, responding to the detailed comments of the reviewers.

We believe that it may be helpful before addressing the specific comments below to precede our responses with an explanation of our approach to qualitative research in general. Many of the comments made by the reviewers in their report are as much about qualitative research in general as they are about our particular study, and so we would like to set out our approach in the round, as context to our detailed comments further below. The open peer review process may be helpful here, as the comments we make are openly available and help to explain our approach, even though not all of the detail can be incorporated in our final paper. This general explanation is reproduced in both our responses to reviewers’ comments, as similar issues arise in both.

Our general approach in responding to the comments of the reviewers on qualitative methods is to try to achieve a balance between, on the one hand, providing clarity and further detail where necessary, and, on the other hand, avoiding over-extending an already long paper. So we have made some changes to the text of the paper with the aim of achieving greater clarity but provide more detailed explanations here in in this report in order to provide fuller explanations that might be too detailed for the paper itself.

Our study uses well-established qualitative research methods. Bazeley (2013) states: "researchers engaging in a qualitative study focus on observing, describing, interpreting, and analysing the way that people experience, act on, or think about themselves and the world around them”. Qualitative research aims to collect and analyse “rich, deep data” (Bryman, 2015) which enable understandings of issues like motivations, beliefs, and values of participants in the contexts in which they act. It focuses on understanding the perspectives of participants, including where disagreement and conflict may exist. Qualitative research typically attempts not just to identify behaviours but also *meanings* associated with behaviours. It is often useful for encountering unexpected issues and influences. Qualitative methods are commonly deployed therefore in studying phenomena and contexts which are emerging, where issues need to be mapped out, and theory developed (which can then in turn potentially be tested in various ways, including quantitatively). We chose to explore the preprints space using these methods in order to gain a rich and deep understanding of the issues involved, specifically including varying motivations, differing behaviours, and conflicting perspectives.

Qualitative data can take different forms and be gathered in different ways, but often take the form of text, e.g. transcripts of interviews, as in our study. In this case the transcripts *are the data* being analysed, and there are well-established methods for carrying out such analysis. Analysis is typically conducted inductively, identifying patterns which emerge from the data. This is normally done through a process like thematic analysis (used in this study) involving a number of steps, as explained by Braun & Clarke (2006), who are cited in our study. There are a large number of other texts that describe this process, albeit with variations e.g. Bazeley (2013), Bryman (2015), Maxwell (2013) etc. In the first stage of the process, the interviews are fully transcribed and then read and reread thoroughly by the researchers. Secondly, the entire dataset is coded – key features of the data being labelled at a detailed level. “Codes…serve as shorthand devices to *label, separate, compile* and *organize* data” (Charmaz, 1983). This is an iterative process involving constant comparisons across the dataset. It can be carried out in various ways, but in our case, we used the NVivo software which facilitates coding of large amounts of text. Coding is a kind of fracturing process, breaking down the data into small fragments, and so the third stage of thematic analysis involves assembling codes into groups or themes. Themes are identified iteratively through constant comparisons, often involved in mapping out themes in relation to one another in order to define and refine them. These were the processes we followed in this study.

In reporting qualitative research, the themes are normally used as a framework, rather than the questions that were initially asked of participants. This is an important aspect of the reporting of an inductive study in order to represent the themes that emerged from the data rather than the initial framework of the questions asked. From analysis of these themes, theoretical insights should emerge. These could take the form of systematic explanations of a context; models of relationships, issues, challenges etc; sets of questions or hypotheses that need to be further investigated; etc. We have tried to provide some of these, albeit tentatively, in our study.

Sampling in qualitative research is normally carried out in a purposive (or purposeful) way. This is described by Bryman (2015):

“Purposive sampling is a non-probability form sampling. The researcher does not seek to sample research participants on a random basis. The goal of purposive sampling is to sample cases/participants in a strategic way, so that the sample are relevant to the research questions that are being posed. Very often, the researcher will want to sample in order to ensure that there is a good deal of variety in the resulting sample, so that sample members differ from each other in terms key characteristics relevant to the research question.”

We describe our sampling approach as a “heterogeneous purposive sampling approach”, which we go on to define as, “aiming to include a wide range of perspectives from actors in the area”. The sample was heterogeneous in a number of respects: it contained representatives of different roles in the scholarly communication system, from different countries (and therefore policy environments), and with different views and levels of experience of using preprints. Participants were selected by us based on an analysis of the field, consultation with our funders and others, and then approached directly and invited to participate. We also advertised on email discussion lists and social media inviting participants, but only selected people to be interviewed who met our sampling criteria. One challenge was in finding researchers willing to be interviewed who were not engaged with preprints, and so in order to do this, we not only used the selection method of approaching people directly but also used “snowball sampling” – asking for recommendations of other people who we might approach from participants. As Bryman (2015) comments:

“Purposive sampling often involves more than one of the approaches outlined… For example, it is quite common for snowball sampling to be preceded by another form of purposive sampling. In effect, the process entails sampling initial participants without using the snowball approach and then using these initial contacts to broaden out to a snowballing method.”

This is what we did, resulting in a final sample of 38.

Sample size is a controversial issue. There is no obvious right answer to the question of, “how many interviews are enough?” The essays addressing that question by well-known qualitative researchers edited by Baker & Edwards (2012) include a wide range of different views, depending on a number of factors. Bryman quotes Mason (2010) as having examined UK and Irish PhD theses using qualitative interviews, which identifies them as using a mean sample size of 31 and a median of 28. Bryman, in his chapter on reporting research, looks in detail at a sample journal article reporting semi-structured interviews which has a sample size of 20 (Jones, Leontowitsch, & Higgs, 2010). Our sample size of 38 is relatively large. One key point to emphasise, however, is that sample size in qualitative research is normally not determined in advance. Rather, the sampling approach will be decided and participants recruited within the sampling frame. The researchers will then make a number of judgements during the data gathering process about when sufficient data has been collected. Chief amongst these is the idea of “theoretical saturation”, where new data “no longer suggests new insights” (Bryman, 2015). This involves a number of judgements, involving a close engagement with the data in relation to the research objectives.

Because qualitative research usually involves identifying areas of motivations, perspectives etc, there is commonly a need to allow participant anonymity. Often interviewees are less frank when they are ‘on the record’. Less frank interviewees tends to lead to a rather bland dataset which makes it difficult to understand the real contours of a landscape. In our study, we gained ethical approval to name participants and their organisations (with their permission) but not to associate any particular quotations with individuals. We have adopted a conventional way of dealing with this by identifying various groups amongst our sample which we do relate with each quotation (“Unengaged researcher”, “Preprint service provider” etc). Naming the group from which the interviewee being quoted came helps to contextualise each quotation, but does not compromise promised anonymity.

Providing extensive extracts of participant quotations is a critical part of presenting findings from qualitative research. Since these quotations are illustrative of the themes identified, and are examples of the data collected, showing them in detail is an important way of demonstrating the validity of the inferences being drawn from the research.

Baker, S. E., & Edwards, R. (2012). *How many qualitative interviews is enough?* Retrieved from http://eprints.ncrm.ac.uk/2273/4/how_many_interviews.pdf

Bazeley, P. (2013). *Qualitative data analysis: Practical strategies*. London: Sage.

Bryman, A. (2015). *Social research methods* (5th ed.). Oxford: Oxford University Press.

Jones, I. R., Leontowitsch, M., & Higgs, P. (2010). The experience of retirement in second modernity. *Sociology*, *44*(1), 103–120. https://doi.org/10.1177/0038038509351610

Maxwell, J. (2013). Designing a qualitative study. In L. Bickman & D. J. Rog (Eds.), *The SAGE handbook of applied social research methods* (pp. 214–253). https://doi.org/10.4135/9781483348858.n7

**Major comments**

***Reviewers’ comments:***

- *Design and Subjects:*
  - *We encourage authors to rely on a more robust review of published literature (including the grey literature) to complement their interviews, as the published literature contains useful context for comments which are obfuscated by the process of anonymous reporting (For example, the sentence, “One preprint service provider observed that only 10% of preprints received comments.” would be more useful to readers if accompanied by a reference with attribution:* *https://asapbio.org/biorxiv* *)*

 

**Authors’ response:**

We have included a literature review in our study and are happy to update it with other relevant literature (as mentioned below and in response to comments of the other reviewers).

We sketch out the literature review and engage with literature again in our discussion, but limit our reporting of our data to doing exactly that – reporting our data. We report therefore that, “One preprint service provider observed that only 10% of preprints received comments” but we have to be careful not to appear to attribute this quotation in a way that makes the interviewee identifiable (see above comments on anonymity). As this quotation is from a “preprints service provider” it is reasonable to assume it is reliable.

***Reviewers’ comments:***

- *Since this work may be of interest to a wide range of readers, we recommend including more detail regarding the chosen experimental design. *
  - *Please provide justification for the experimental design, for example, why were interviews used instead of a survey? *
  - *What are the methodological considerations that would be helpful for readers outside of qualitative research to understand: limitations, constraints, advantages over more quantitative methods? *
  - *Please define semi-structured interviews — how were researchers encouraged to expand on thoughts? *
  - *Please define “purposive heterogeneous sampling approach.”*
  - *Please clarify why the sample size is 38.*

**Authors’ response:**

To respond to the above bullet points in turn:

Justification for the approach. As the above general explanation outlines, we chose qualitative methods in order to explore in-depth the “varying motivations, differing behaviours and conflicting perspectives” of key actors in the preprints space – something qualitative methods are often designed to do. We have added some more detail to the method to clarify this, notably in the first paragraph of the Methods section:

“As preprints and preprint servers are still innovative developments for most disciplines, it is important to gain an in-depth understanding of the perspectives of different stakeholders. In order to explore issues, such as varying motivations, differing behaviours, and conflicting perspectives, particularly in emerging areas, qualitative research methods are often deployed, since they are well suited to such investigations. We chose to carry out detailed interviews of key actors in this space who could explain in depth their perceptions, attitudes and practices in relation to preprints…” etc.

Limitations and constraints. We have also added a new section in the Methods section on “Limitations and constraints”. The main limitation with qualitative research is lack of generalisability. Whilst results may be *transferable* to other contexts, generalisability is not possible from qualitative research alone. Qualitative research is designed to explore specific instances of phenomena and generate theory and hypotheses which might then be tested using other methods, including quantitative methods where findings might be generalised. Our new section reads:

“Limitations and constraints. Like many kinds of qualitative research, this study was designed to be exploratory; in this case, to map out key aspects of the preprints space and suggest policy responses.  Our conclusions are tentative. Many of interviewees were selected because of their knowledge of the issues under investigation, and although our findings based on their views may be transferable to other contexts, they cannot be generalised without further testing, as with most qualitative research. There was a bias in our sampling of participants aware of and engaged with preprints. Further research, using other methods, will be needed in future in order to generalise across communities as a whole, including non-engaged researchers. Furthermore, some stakeholder groups, such as publishers (who only have very limited representation in this study), and other groups (such as non-academic users of the research literature) could usefully be included in future studies. Our coding was undertaken using agreed protocols and involved a process of validation provided by three different members of the authorial team but necessarily involves interpretation and judgement on the part of the researchers.”

Semi-structured interviews. Semi-structured interviews have ‘spine’ of common questions running through the interview shared by all participants (or all participants in a particular group), but allow room for the interviewer to pursue areas of interest arising from participant responses, including probing for greater clarity, where needed. It is probably the most common interviewing approach for qualitative research. We have added a brief explanation to the paper and also a reference to a more detailed explanation should the reader be interested to follow it up:

“Interviews were conducted using a semi-structured approach – incorporating a ‘spine’ of common questions for all participants, and some questions specific to different actor groups – allowing room for the interviewer pursue areas of interest arising from participant responses, including probing for greater clarity, where needed (Bryman, 2015).”

Sampling approach. We have described purposive heterogeneous sampling above in our introductory explanation. We have expanded the explanation in the paper itself to clarify this.

“The study adopted a heterogeneous purposive sampling approach, aiming to include a wide range of perspectives from actors in the area, selected in a “strategic way” in order to address the objectives of the study (Bryman, 2015). The sample was heterogeneous in a number of respects: firstly, it contained representatives of different roles in the scholarly communication system; secondly, it included participants from different countries (and therefore policy environments); thirdly, it comprised interviewees and with different views and levels of experience of using preprints. Participants comprised senior representatives from research funders, research-conducting organisations (universities and research institutes), preprint services, other related service providers (such as infrastructure providers), as well as researchers, both researchers demonstrably engaged with preprints (they had themselves posted a preprint) and non-engaged (there was no evidence of them having posted a preprint)…” etc.

Sample size. Sample size is addressed in our introductory explanation. 38 is a relatively large number for a study of this kind, enabling the study to achieve the desired heterogeneity and theoretical saturation. We have added a sentence explaining this in the Method: “The sample allowed the study to achieve the desired heterogeneity of actors and perspectives.”

***Reviewers’ comments:***

- *Please provide more information about the subjects to help readers assess relevance:*
  - *What is their geographical context?*
  - *How knowledgeable were subjects, and do they bring bias for or against open science into their statements? *
  - *How did interviewees arrive at their opinions? How much close experience do they have with preprints? Were participants asked to answer only within their own expertise? *

 

**Authors’ response:**

All of our participants and their affiliations are named and so readers can draw judgements from this – this information is available in the extended data. As already discussed, we have an ethical commitment not to associate individuals with specific quotations, which is conventional for research of this kind as explained in the introductory explanation above.

***Reviewers’ comments:***

- *There are quoted statements that reflect inaccurate understandings and are not always qualified by the author: for example, “relating to whether preprints are seen as an appropriate object for evaluation in exercises such as the UK’s Research Excellence Framework, about which there remains ambiguity. One representative of a university stated: “they’re not acceptable for REF, so they’re not even part of the equation. So it’s the author’s accepted manuscript is the currency we deal with.” (Research performing organisation)”  This is inaccurate and misleading (see* *https://www.ref.ac.uk/publications/guidance-on-submissions-201901/* *) but there is no further commentary from the authors. Please add clarification that the interviewee responses are not necessarily factual. (It would be interesting to compare interviewee opinions with known facts to understand which topics are well-understood.)*

 

**Authors’ response:**

On the specific issue of the REF and preprints, the guidance was been updated at the beginning of 2019 whilst we were still undertaking our interviews. We have added a comment in the paper clarifying the situation, but acknowledging there is still ambiguity in this area in terms of practice: the official rules of the REF and actual practice may often be in tension:

“The concept of “standing” (Neylon et al., 2017) is relevant here, with, as an example, standing relating to whether preprints are seen as an appropriate object for evaluation in exercises such as the UK’s Research Excellence Framework, about which there had apparently been uncertainty. One representative of a university stated:

*“they’re not acceptable for REF, so they’re not even part of the equation. So it’s the author’s accepted manuscript is the currency we deal with.” (Research performing organisation)*

The REF guidance was updated in January 2019 (whilst our interviews were still ongoing) and now states that preprints can be included in REF submissions, but expresses a preference for “final versions” of papers rather than preprints (Research England, 2019). It is, however, also possible that institutions have developed local conventions for REF submission which in fact discourage submission of preprints, as might be inferred from this quotation.”

In some ways, there is a balance to be achieved here. Qualitative research aims to find out about perceptions and this can include misunderstandings. It is important to report these, as misunderstandings often shape behaviours. The extent to which the researcher should correct the participants in their reporting is a moot point in general. That being as it may, in this case, we do recognise there was a need to comment.

***Reviewers’ comments:***

- *Clarity of presentation: *
  - *Please provide more quantitative context to the opinions expressed. For example, for phrases such as “Some researchers” it would be useful to be more specific. Please also be more quantitative in assessment of the themes — it is useful to know if a sentiment was expressed by a majority or minority, as shown clearly in tables of benefits and challenges. Please provide aggregate data of thematic analyses, not just quotes, and use specific numbers and proportionality (Two of Five researchers mentioned X as a benefit).*

**Authors’ response:**

We have addressed this comment by amending wording to be as precise as possible within the constraints of a qualitative study. Once again, there is a balance to struck here. On the one hand, as a general principle, it is essential to recognise that qualitative research should not be analysed quantitatively. Inferences cannot be drawn from the data in any statistically valid way from quantitative analysis of a dataset like this, and it is important not to give the impression that they can. Certainly, including percentages would not be appropriate. In any case, the data is not sufficiently structured to allow for reliable identification of a type of response which could be reported quantitatively. Every datum in a qualitative dataset is actually unique and although it is possible to identify commonalties, it is not normally possible to say that a certain proportion of participants said one thing or another. This likely to lead inappropriate homogenisation of views.

Furthermore, it is important not to confuse prevalence with significance. In a qualitative dataset, some ideas may be mentioned by a large number of participants, but this does not necessarily mean they are significant. Significance may not, of course, be easy to judge (and it is a matter of judgement based on careful analysis) but it is not a simple issue of numbers of times an issue is mentioned in the dataset. We have however made some attempts to identify prevalence of issues raised in discussing pros and cons of preprints, something which is possible (simply identifying topics mentioned), with the relevant caveats.

On the other hand, it is perfectly reasonable to mention some quantitative terms, “the majority”, or “a minority” or if an issue was only mentioned by one or was mentioned by all participants. That is what we have tried to do within the bounds of what is defensible.  

***Reviewers’ comments:*** 

- *The paper combines elements of a literature review, white paper/policy piece, and a qualitative study. It would be helpful to clarify the origin of ideas by separating the work clearly into Results and Discussion sections. (As one example, under the heading “Financial sustainability and business models,” it is unclear whether the proposed taxonomy was expressed by interviewees or generated by the authors. Similarly, it is unclear whether use of the term “seminal” comes from interviewees or authors.)*

**Authors’ response:**

This point is well made. The systematisation of the different models or scenarios of delivery of preprint services is ours based on a composite analysis of our data. Many of our participants talked about ownership and sustainability issues but none of them organised their remarks in this way. We had thought the systematisation of views helped to contextualise the reporting of the data but it does create the potential for confusion, as the reviewers point out. So we have, therefore, pulled this section out of the findings and added it into the discussion in order to make it clear that it is our analysis arising from the data not a direct reporting of the data.

***Reviewers’ comments:***

- *Readability:*
  - *Please use active voice and concise language to improve readability.*

 

**Authors’ response:**

We are conscious that conventions vary in this area across disciplines or even journals. Many life sciences researchers have moved over recent years to using the active voice. However, we were not aware of there being a house style for F1000. The discursive style we have adopted using a combination of active and passive voice is conventional for the reporting of the research we have carried out. We have, however, made some adjustments to improve clarity.

***Reviewers’ comments:***

- *Bring interview questions and list of participants into this report — it is hard to interpret findings while having to continuously refer back to other sources, e.g. the 2019b interview structure paper.*

**Authors’ response:**

We believe the comments from the two sets of peer reviewers are somewhat in tension with regard to the level of detail that should be included, with the other reviewers stating:

*The reviewers wonder if the results section as a whole, which is relatively long in comparison to the rest of the paper, should be limited to Table 5 and its description and avoid the unending narrative of anecdotal specific responses.*

Given the insertion of the interview questions and list of participants would make the paper still longer, we believe it remains appropriate to make them available as separate, citable objects which are referenced by the paper.

Finally, sharing interview questions and participants via ‘extended data’ is required by the F1000Research Author Guidelines, which state that: “F1000Research does not accept supplementary material. Additional materials that support the key claims in the paper but are not absolutely required to follow the study design and analysis of the results, e.g. questionnaires, or supporting images or tables, can be included as extended data.”

***Reviewers’ comments:***

- *Claims: *
  - *The abstract sets the reader up to expect a review of preprint servers that have emerged since 2013. However, the methods section discloses that coverage of the study is limited to biology, chemistry, and psychology. It would be helpful to readers to state this up front. It is also unclear how this scope was applied in the selection of study subjects. It is difficult to determine if the scope influenced the selection of participants as listed in* *https://zenodo.org/record/2654832#.XTeUB5NKjs0* *– the questions listed at* *https://zenodo.org/record/3240426* *do not seem specific to these three disciplines and the quoted answers in this report suggest interviewees’ responses were not limited to these three disciplines. Please clarify how the scope of the interviews was constrained to the stated subjects, or if not, please revise the stated scope to more accurately reflect the scope employed.*

**Authors’ response:**

We have changed the text at the beginning of the methods section to provide greater clarity about our approach, as follows:

“We chose to carry out detailed interviews of key actors in this space who could explain in depth their perceptions, attitudes and practices in relation to preprints. Participants were asked about their perspectives on preprints in general, but we intentionally recruited interviewees (where they had disciplinary affiliations) particularly from disciplines where preprint services are relatively new and rapidly growing. These were biology, chemistry and psychology, corresponding to preprint servers, bioRxiv, ChemRxiv and PsyArXiv. Focusing on these areas helped us to gauge the impact that preprints are having in areas where they are more innovative, and since many of our participants were able to speak more generally about preprints, we were able to draw comparisons with disciplines where preprints are established and which are better represented in the literature (e.g. physics, computer science, and economics).”

It is important to note that our study was not designed to elicit comments about specific servers in particular but rather about participants views on preprints in general. Their own experiences, based as some of them were in particular disciplinary communities, will, of course, be coloured by their familiarity with particular servers, but we wanted to explore their views on preprints more generally than that.

***Reviewers’ comments:***

- *The abstract does not reflect the content. The interview responses are summarised in general terms, not related to defined scope or the newer servers. A substantial proportion of this article is review material: either in the explicit literature review section, or as author-contributed content interspersed with summaries of interviewee responses. For example, conclusions listed in the abstract appear to be drawn from the literature review.*

 

**Authors’ response:**

We have made changes to the abstract that we hope correct this, including ensuring findings reported relate closely to the data (although for the most part these do correlate with the literature).

***Reviewers’ comments:***

- *“Our study is the first using empirical data to understand the new wave of preprint servers” — this is not supported by the study content: no results are specifically related to the newer servers identified in the introduction; quoted excerpts indicate that interviewees responded in general about all servers and disciplines. Furthermore, prior surveys and bibliometric analyses have looked at a subset of these servers, either individually or in groups (eg* *https://osf.io/5fk6c/* *).* *^1^*

 

**Authors’ response:**

We have made changes to the abstract that we hope correct this.

**Minor comments**

***Reviewers’ comments:***

*We recommend making the following amendments to improve the clarity and accuracy of the report.*

*In the Introduction and literature review: *

- *The summary of the longer history of preprint efforts across disciplines, would benefit from incorporating and citing ‘The Brief Prehistory of Preprints” (Cobb, 2017)* *^2^*

**Authors’ response:**

Added.

***Reviewers’ comments:***

- *Zuckerberg Foundation should be corrected to Chan Zuckerberg Initiative.*

 

**Authors’ response:**

Corrected.

***Reviewers’ comments:***

- *Discussion of new servers would be improved with a note as to their current size, and with awareness that not all new ‘preprint’ servers are exclusively for preprint content (many COS servers include postprints that are not possible to easily distinguish from preprint versions).*

**Authors’ response:**

We have added figures for bioRxiv, ChemRxiv and PsyArXiv.

***Reviewers’ comments:*** 

- *“This paper aims to explore…by investigating current practices, drivers and barriers to [preprint] use.” The interviews indicate perceptions and attitudes but do not reveal actual behaviours or practices. Please clarify how this study examines current practices.*

 

**Authors’ response:**

The data covers behaviours and practices in a number of ways. Most prominently, the data indicates differences in uptake of preprints by researchers, with some being engaged and talking about their experiences and the positive outcomes that have accrued in their view. Others were sceptical and spoke about their lack of engagement based on their views of the some of the challenges presented by preprints. The thematic area covering perceived benefits and challenges has experience of adoption (or non-adoption) running through it. The theme of “Disciplines, cultures and practices” also has behaviours and practices throughout.  In it, we refer to differing levels of awareness and “adoption”, “a willingness to experiment” etc. and present illustrative quotations from interviews. The earlier section on definitions also has some elements of behaviour, with one participant, for example, talking about posting a preprint when it was “submission ready for a journal”. The behaviours and practices are then closely linked to and shaped by the perceptions and attitudes, as might be expected. We have tried to make this clear at the beginning of the sub-section on benefits and challenges: “Participants highlighted a number of (potential) benefits and challenges of preprints which were seen to relate to particular practices around adoption or non-adoption of preprints”. 

***Reviewers’ comments:***

*Figures and tables:*

- *Fig 1: To increase accessibility, please provide as raw text with headings or tags to indicate which stakeholder group you believe is most relevant*

**Authors’ response**

Figure 1 is now available in csv format (text only) via Zenodo. This is referenced in the text as Chiarelli et al 2019c - http://doi.org/10.5281/zenodo.3539032.

***Reviewers’ comments:***

*Literature review:*

- *The authors focus on four main issues — how were these arrived at? Was the literature review systematic in any way? *

**Authors’ response**

We did not perform a formal systematic literature review but our reading of the literature is that these issues emerge. We have added a new category to cover the more empirical studies now emerging, mostly in 2019.

***Reviewers’ comments:*** 

- *Given the emergent phase of preprint infrastructure in biology, chemistry and psychology, and as the authors note that empirical data is lacking, it may be equally valuable to include more grey literature (blogposts, webpages) in this review, given that several of the cited articles are opinion pieces, albeit as editorials or peer-reviewed review articles. Of note, we find personal blogs are a useful source of stories that detail benefits and drawbacks of preprints from personal experiences.*

 

**Authors’ response**

We have included some of these more informal sources, including information form the ASAPbio web site, Scholarly Kitchen blog, SciELO blog and numerous news sections of journals. We have added an example of an exchange on Twitter. We do not claim to be exhaustive but have been intentionally selective to represent the main strands of debates. Including many more would make an already long article even longer when we would prefer the main focus of the article to on the data we present.

***Reviewers’ comments:***

- *"Dis-benefits:" is not a common term, perhaps use “drawbacks,” “concerns,”, “disadvantages” or “potential risks”.*

**Authors’ response:**

We have changed the term to “challenges”.

***Reviewers’ comments:***

- *When discussing the lack of quality assurance, the authors seem to make an implicit assumption here that journal-led peer review assures quality. It would be fair to contextualise this section with some literature on peer review to understand variety of peer review outcomes on quality of paper and validity of findings.*

**Authors’ response:**

We have added reference (Lee, et al., 2013) in the literature review on problems with peer review. Concerns about quality were raised by our participants, which we report, although as we also say, many were eager to report the imperfections of peer review as well.

***Reviewers’ comments:***

*Results:*

- *In the discussion of preprints in the medical field, please note that the concerns raised are being addressed by medRxiv.* *https://www.medrxiv.org/*

**Authors’ response:**

medRxiv had not been released when our data was collected. Our data are inevitably a snapshot from the time it was collected. However, we note that the quotation from this area in the paper covers many of the checks now being carried out by medRxiv.

***Reviewers’ comments:*** 

- *In the table of business models: *
  - *Note that bioRxiv uses 3rd party technology, Highwire.*

**Authors’ response:**

Corrected

***Reviewers’ comments:***

- *“Publisher driven” models are not new; PeerJ is older than bioRxiv, Nature had Nature Preceedings in the early 2000s, and bioRxiv has had J2B preprints since its inception. *

**Authors’ response:**

Noted and details about the closure of PeerJ preprints also now added.

***Reviewers’ comments:***

- *How does the outsourcing of infrastructure to a third party enable sustainability? Arguably, depending on the third party, this leaves the service vulnerable to changes in service entirely outside its control.*

**Authors’ response:**

We have added clarification on this, as follows:

“Model 2 is a version of 1 in which some infrastructure is outsourced to a third party, something which might help to enable sustainability by creating efficiencies through economies of scale (a common benefits of outsourcing). The infrastructure provided by the Center for Open Science is an example of such benefit, with multiple preprint servers being run by the same organisation and on the same infrastructure, avoiding duplication, and therefore creating efficiency.”

***Reviewers’ comments:*** 

- *Individual authors can still submit to PeerJ.*

**Authors’ response:**

We have updated references to PeerJ following the announcement of their closure.

***Reviewers’ comments:*** 

- *Most publisher-mediated preprint submissions are voluntary for authors (a notable exception being F1000).*

**Authors’ response:**

We have added a note to this effect in the Introduction.

***Reviewers’ comments:*** 

- *Regarding the statement of subject preferences: we have conducted a survey on these preferences that may provide additional context:* *https://asapbio.org/asapbio-funders-workshop* 

**Authors’ response:**

We have reviewed the survey results at the URL cited and agree they are useful and interesting. However, we cannot see substantial information about “subject preferences” in the data. We have not therefore made any changes in response since we are unsure what the recommendation of the reviewers refers to.

***The following suggestions may also help improve the paper but are not essential.***

***Reviewers’ comments:***

*In the Introduction and literature review:*

- *Open Research platforms could be discussed in context of F1000Research, since this is the model they are derived from (in collaboration with F1000). *

**Authors’ response:**

The Open Research Central services are further examples of the model developed by F1000. Since we are not attempting to be exhaustive in listing of services but are rather giving examples, and because Open Research Central uses the same model as is “powered by” F1000, we would prefer to keep our introduction at the length it is without giving more examples, which would further extend it.

***Reviewers’ comments:***

- *Please clarify the age of PeerJ and* *Preprints.org* *in comparison with bioRxiv.*

**Authors’ response:**

We have done this, adding a note of the launch (and closure) of PeerJ in the opening paragraph.

***Reviewers’ comments:***

- *PLOS is not the only journal that deposits preprints to biorxiv using the J2B mechanism — several do, please refer to the list of journals offering direct transfers to bioRxiv (* *https://www.biorxiv.org/about-biorxiv* *).*

**Authors’ response:**

We have done this, adding a reference in the opening paragraph.

***Reviewers’ comments:***

- *Regarding trust in preprints, it would be relevant to mention or cite the current effort by COS to better understand indicators of trust in preprints:* *https://cos.io/about/news/center-open-science-receives-grant-alfred-p-sloan-foundation-study-trust-and-credibility-preprints/* *.*

 

***Reviewers’ comments:***

*Figures and tables:*

- *Fig 1 introduces some interesting questions, however many of these are related to topics that are brought up for the first time in this figure.*

**Authors’ response:**

Yes, ours was an exploratory study which had as one of its aims to identify areas requiring further work. Our data pointed to a wide range of questions which now need to be pursued in order to understand more about the (potential) future of preprints. What we have done is to help to provide clarity around what the they key questions are and how they might begin to be addressed (e.g. which actors should be involved in addressing them). This was designed to be helpful to policy makers in particular.

***Reviewers’ comments:***

- *Table 6: The concern of reputational damage is indeed in the literature; please see point 5 here:* *https://smallpondscience.com/2017/07/24/whats-up-with-preprints/* 

**Authors’ response:**

Updated

***Reviewers’ comments:***

*Literature:*

- *“However, Bourne et al. (2017) controversially extend the definition to include “a paper that has been peer reviewed and…was rejected, but the authors are willing to make the content public”.” — Please provide reasoning or citation as to why this is controversial.*

**Authors’ response:**

The context as a whole is important: “For (3) versioning, the relationship of a preprint to peer review is central. Desjardins-Proulx et al.’s (2013, p. 1) observation is typical in stating that preprints are made available, “before, or in parallel to, submitting them to journals for traditional peer review”. Suber (2012, p. 102) points out that this is not to “bypass peer review”, but that it applies to “works destined for peer review but not yet peer reviewed”. However, Bourne et al. (2017) controversially extend the definition to include “a paper that has been peer reviewed and…was rejected, but the authors are willing to make the content public”.

The point is that if preprints are defined as papers “destined for peer review” then including papers was rejected in a peer review process extends the definition and does so in a controversial way because it might imply a by-passing (or ignoring) of the peer review process. We note that in our data some participants questioned whether an output can be considered a preprint if it is not ultimately formally published.

***Reviewers’ comments:***

- *Accessibility — note that preprints are not necessarily open access, where they are not provided with permissive licensing, and this is certainly the case for bioRxiv.* *^3^*

**Authors’ response:**

Point noted. We have changed the description in Table 1 to, “Preprints are openly available online”. In the associated discussion we talking about availability of content but do not use the term ‘open access’.  This was intentional, as it allows us to side step the question of definitions of open access, which is a controversial issue (gratis, libre etc). We appreciate the point about licensing and this was one issue mentioned in our dataset and to which we make reference.

***Reviewers’ comments:***

- *One potential benefit to ECRs that is not mentioned here is being able to demonstrate productivity to funders and hirers. This would be useful to append to the current paragraph, given it is raised in the interview questions.*

**Authors’ response:**

Good point, and as this is a somewhat different point than achieving visibility, we have added it to the paragraph:

“Early dissemination is seen by some as especially useful to a number of members of the scholarly community in particular, with early career researchers (ECRs) commonly identified as specific potential beneficiaries, as preprints can allow them to rapidly achieve “visibility” and demonstrate productivity in job and grant applications (Desjardins-Proulx et al., 2013; Sarabipour et al., 2018; Tennant et al., 2019).”

***Reviewers’ comments:***

- *“There also appears to be little acknowledgement that the claim stands in tension to the one cited earlier that preprints often differ little from final published versions.” — There is only tension if both claims concern all preprints. It is possible for there to be preprints that improve through a lot of feedback as well as preprints that undergo no/very little change.*

**Authors’ response:**

We have updated this sentence to read “There also appears to be little acknowledgement that the claim, *when used to make the case for preprints in general terms*, stands in tension to the one cited earlier that preprints often differ little from final published versions.”

***Reviewers’ comments:***

- *Please use unshared or unpublished instead of “homeless”.*

**Authors’ response:**

“Homeless” has been used elsewhere in the context of preprints (e.g. Berlin, 2018) and more generally in the context of scholarly communication. Mega-journals have been described as “allowing for the publication of research that might not find a home in traditionally more selective journals” (Wakeling, et al. 2017). That definition helps to make the point that “homeless” does not just mean outputs that are unshared or unpublished but ones that might not otherwise find a venue for publication. We therefore propose to retain the term.

Berlin, S. (2018). If the papers don’t come to the journal…. *EMBO Reports*, *19*(4). https://doi.org/10.15252/embr.201845911

Wakeling, S., Spezi, V., Creaser, C., Fry, J., Pinfield, S., & Willett, P. (2017). Open access megajournals: The publisher perspective (Part 1: Motivations). *Learned Publishing, 30*(4), 313–322. https://doi.org/10.1002/leap.1118

***Reviewers’ comments:***

- *We recommend avoiding the term “policy stack,” as there is no dependent relationship among these different groups/stakeholders.*

**Authors’ response:**

We have removed the term from the paper.

***Reviewers’ comments:***

*Methods:*

- *This section ignores the efforts of researchers as change agents.*

**Authors’ response:**

We have now acknowledged the potential for change agents to affect community norms within our description of the ‘nature of the social system’.

***Reviewers’ comments:***

*Results:*

- *Please provide evidence for this statement: “we note that adjacency between disciplines appears to be playing an important role.” *

**Authors’ response:**

We have adjusted the wording of this to make sure it is clearly defensible:

“Finally, we note that adjacency between disciplines may play an important role. For example, disciplines close to those which posted preprints (e.g. those close to some areas of computer science or physics, which use arXiv consistently) may be more favourably disposed to the practice compared to those from other disciplines.”

The extension of arXiv to other adjacent disciplines is evidence of our point and we see hints of this elsewhere – but at early points.

***Reviewers’ comments:***

- *“Many of the participants agreed that preprints servers could be a useful outlet for otherwise ‘homeless’ research outputs, even though this goes counter to the emphasis of many of preprints as early versions of outputs later formally published elsewhere.” — this attitude may reflect a lack of publication venues for such content, not a lack of desire to publish these other outputs in a peer-reviewed destination.*

**Authors’ response:**

Yes, agreed (see our remarks on “homeless” above). It does shift the model of preprints as being early versions of papers that are later formally published elsewhere, however.

# Peer review: Reviewer response for version 1

**DOI:** 10.5256/f1000research.21512.r50461

**Published:** 2019-07-23

**Authors:** Sarvenaz Sarabipour, Humberto Debat

**Affiliations:** 1 Institute for Computational Medicine, Department of Biomedical Engineering, Johns Hopkins University, Baltimore, MD, USA; 2 Center of Agronomic Research, National Institute of Agricultural Technology (IPAVE-CIAP-INTA), Córdoba, Argentina

**Summary:**

 In this manuscript Chiarelli *et al.* assess preprints from the point of view of a diverse group of stakeholders. The authors perform an analysis based on 38 interviews with representatives of research funders, research performing organizations, preprint servers and service providers; and researchers (engaged and unengaged). Additional discussion is provided on the benefits and challenges of preprint posting, along with issues such as infrastructure and financial sustainability and the definition of a ‘preprint’ in different communities, and the impact this has on further uptake. This study provides a thorough investigation on an emerging and relevant topic, giving a platform to the opinions of under-represented stakeholders on the discussion of preprints: a key driver of the transformation of the scholarly publishing landscape.

**Comments:**

 We would like to ask the authors to address the following:

 The introduction may benefit from additional literature to balance the many sections. For instance:

 1. On Page 3: “However, skeptics have questioned the value of preprints and even suggested they may be dangerous – circulating versions of articles before they have been quality controlled by peer review may lead to unnecessary risk, particularly in disciplines like medicine (Sheldon, 2018).” The only negative note on preprints by Sheldon 2018 was well rebutted by at least 5 publications:

- Maintaining confidence in the reporting of scientific outputs - Sarabipour *et al. *(2018) ^1^.
- Preprints are good for science and good for the public - Sarabipour (2018) ^2^.
- Preprints help journalism, not hinder it - Tennant *el al. *(2018) ^3^.
- Together scientists and journalists can spot poor preprints - Fraser & Polka (2018) ^4^.
- Preprints: recall Nature’s nasty past - Cobb (2019) ^5^.

Articles 1-4 above address the relationship between scientific reporting and journalism. Further to the authors remark on “unnecessary risk particularly in disciplines like medicine” - preprint servers carry a highly visible note on preprinted manuscript stating that these research products are not peer-reviewed.

 Regarding biomedical and medical preprints and risk to public: This is not a compelling argument since care is delivered to patients by physicians. MedRxiv was recently established and is accepting manuscripts to accelerate medical research. The server front page notes that: "Caution: Preprints are preliminary reports of work that have not been peer-reviewed. They should not be relied on to guide clinical practice or health-related behavior and should not be reported in news media as established information.”

 2. On Page 3: “the current and potential future role of preprints as a vehicle for scholarly communication” - there are and will be many role(s) for preprints in the current and future of the scholarly endeavors and they are summarized here:

- On the value of preprints: An early career researcher perspective - Sarabipour *et al.*(2019) ^6^
- https://asapbio.org/reading
- In praise of preprints - Fry *et al*. (2019) ^7^.

3. On Page 4: “much of the literature is still to be found in editorials and opinion pieces rather than data-driven research” - the fact is that the number of data driven literature on preprints is growing rapidly and are noteworthy. Examples are:

- Tracking the popularity and outcomes of all bioRxiv preprints - Abdill & Blekhman (2019) ^8^.
- Releasing a preprint is associated with more attention and citations - Fu & Hughey (2019) ^9^.
- The effect of bioRxiv preprints on citations and altmetrics - Fraser *et al.* (2019) ^10^.
- Comparing quality of reporting between preprints and peer-reviewed articles in the biomedical literature - Carneiro *et al.* (2019) ^11^.

4. On Page 4: “Accessibility (4) is crucial, with a preprint normally defined as being (or assumed to be) openly available: it “can be viewed without charge on the Web” and “Therefore, the venue for distribution of preprints is often assumed to be a freely-accessible server of some kind”. It is a fact that all preprint servers are freely accessible to any human being that has access to internet around the world: free of charge to submit by authors, free of charge to read by all anywhere on the planet.

 5. On Page 5: “To say that an output is “deserving” of dissemination is, of course, a value judgement and difficult to demonstrate for each deposit as it is made, but it is one that is implicit in much of the discourse on preprints.” - this is not entirely true. The value of preprints are well known/articulated/discussed at this point as preprints accelerate research and altmetric attention to scientific work: Please see references 8-11 above. Preprints have been deposited on arXiv for nearly three decades now and are invaluable to the physical sciences community. Other forms of pre-peer review material such as computer code and protocols have been disseminated open source and their value is well noted in various fields.

    

 6. On Page 5: “dis-benefits of preprints” - unless the authors may provide any evidence of “dis-benefits of preprints” besides one note (Sheldon 2018), it is best to avoid using this word “dis-benefits” and consider rephrasing to “perceived dis-benefits”. Please see reference 1-5 above.

 7. On Page 5: “Early dissemination can be useful to some particular members of the scholarly community, with early career researchers (ECRs)” - preprints and all other forms of scholarly pre-publication material are beneficial to ALL researchers not jut ECRs. Timely release of scientific results in the form of preprints and other forms such as code and data bases have already accelerated research by encouraging sharing of ideas, resources and as discussed in previously suggested references above.

 8. On Page 5: “Perhaps the most prominent criticism of preprints relates to this last issue: the lack of quality assurance through peer review (Sheldon, 2018). As well as a general concern about lowering quality standards, lack of quality control has been seen as potentially dangerous as “reports that have not undergone formal peer review [organised by a journal] could be misleading” (Lauer *et al*., 2015). Furthermore, uncertified science might be reported prematurely in the media and might even give rise to ‘fake news’ (Sheldon, 2018). Some insist that, at the very least, the opportunity to disseminate knowledge rapidly without peer review may encourage academics to produce low-quality outputs on fashionable topics (Teixeira da Silva, 2017).” The authors should declare that there are no evidences supporting all these bold statements. The Sheldon (2018) news article in fact explicitly states its speculative nature in the title which reads “preprints could promote confusion and distortion”. Please see references 1-5 above.

 9. On Page 5: “Others have gone further and questioned the value of self-appointed reviewers, as opposed to those selected by journal editors (see the issue of “self-policing” highlighted by Harnad, 1998).” This is a misleading claim. Voluntary reviews of preprints are done by researchers in that field or community and are not self-appointed by the authors. The open access nature of preprints allows the potential assessment of manuscripts by more than 2-3 (more than journal reviewers) normally and there is no evidence on these reviews having different quality than journal peer-reviews.

 10. On Page 5: “Preprint posting, however, is unlikely in any case to substitute for the valuable role played by selective journals in filtering content”. This is an unfounded claim as a system of preprint servers and overlay journals may very well replace the outrageously expensive and time-consuming system of for profit journal publishing in under a decade. Please see reference 12.

- A proposal for the future of scientific publishing in the life sciences - Stern & O’Shea (2019) ^12^.

11. On Page 5: “Whilst this convention has come under criticism and been withdrawn by some publishers, it still exists for some journals, e.g. in medicine and chemistry (Lauer *et al*., 2015; Teixeira da Silva & Dobránszki, 2019)” - this sentence is misleading. In the current scenario, authors should consider changing “some” to “most” publishers.

 12. On Page 6: “For example, it is not universally agreed when an output should be citable (in the literature, funding proposals or promotion cases)”. It is now becoming more and more agreed upon that preprints should be cited in articles and research grant proposals. Major funding bodies such as NIH, CZI and Wellcome Trust allow and encourage citation of preprints. Many journals allow citation of preprints and other research outputs (eLife and PLOS family of journals are examples).

  13. On Page 6 and other pages: “Scooping”, and “a claim of precedence”: Scooping is a perceived concern of researchers and is unfounded. Many researchers present unpublished research at conference. Perceived preprint concerns and claim of precedence have been discussed in reference 6 above.

 14. On Page 6: “or when it can be used to establish a claim of precedence” and “In disciplines where a preprint is not considered appropriate to establish precedence, it has also been suggested that making a preprint available may actually encourage research to be scooped by rival researchers who publish in a recognized journal before the preprint authors (the ‘flip side’ of the priority claim argument above) (Kaiser, 2017).”Regarding these repeated sections on scooping and priority claims:

- 2-3 papers published via preprints or journals in close temporal proximity of each other have all been projects 2-4 years in making so it is very difficult to assess who did it first.
- Who gives researchers priority? A preprint that is posted online has a date and that gives precedence to the work. In reality, as researchers we all know that priority is given to researcher by their field and their peers. Scientific research is often presented at conferences well in advance of preprints or journal publication. As we know what matters in research is scientific reputation and that is given to researchers by their peers and field during a career in scientific research.
- Multiple papers published via preprints or journals in close proximity of each other only show the reproducibility of one another are need to be celebrated not feared so repeated arguments on scooping and priority claims do not move a positive conversation on research forward specially for early career researchers and the future generations of researchers.

15. On Page 6: “Some have argued that initiatives such as Declaration on Research Assessment (DORA), with its emphasis on the quality of the output rather than venue of publication, promote use of preprints (Polka, 2018)”. It is noteworthy that DORA has now become a major international initiative and is signed by over 1,400 organizations including major universities and other research institutions and 14,000 researchers.

**Other comments to the MS text:**

 A) The Title:  Authors could consider an alternative title “Preprints and Scholarly Communication: a preliminary/exploratory study on Adoption, Practices, Drivers and Barriers”

 B) The Abstract:

- “Our study is the first using empirical data to understand” - please change to: Our study **uses** empirical data… (See comments below highlighting other empirical studies on the topic).
- “The main concerns are related to the lack of quality assurance and the ‘Ingelfinger rule’.” Please change to: main **perceived **concerns.

C) The Introduction:

- On Page 3: “key actors”. It is puzzling how the authors determined which the key stakeholders are in the preprints ecosystem. Authors should discuss why they did not incorporate “scientific journal publishers” as a relevant actor for this work. It is interesting that they incorporated “publishers” as node in Figure 1 as a stakeholder group but failed to incorporate “representatives” during their interviews.
- On Page 3: “(Carà et al., 2017) .” Please delete the space.
- On Page 3: “The study is the first using empirical data”.  Please see our comment on the abstract.
- On Page 3: “Zuckerberg Foundation“ - the correct name is the Chan-Zuckerberg Foundation (CZI).
-  On Page 3: “The study is the first using empirical data”. Please see the comments on the abstract above.
- On Page 4: “…relatively small number of peer-reviewed studies focusing on preprints – much of the literature is still to be found in editorials and opinion pieces”. The authors surely know that in general opinion pieces published in scientific journals are also peer-reviewed.
- “Defining preprints. Key components. Table 1”. A relevant aspect of preprints that is not contemplated here is that preprints are published open access free of charge. Perhaps that important feature could be incorporated in component 4. Maybe this feature, highly relevant for unprivileged research communities, was neglected given the sampling strategy employed (see comment below).
- On Page 8: “The study adopted a **heterogeneous** purposive sampling approach, aiming to include a wide range of perspectives from actors in the area. Participants comprised **senior representatives**…”. If the authors selected senior representatives of each stakeholder categories, one could question how heterogeneous the sampling approach was. Moreover, authors mention the use of snowball sampling, which is linked to homogeneous sampling. In addition, participants correspond to only eight OECD High-income economies according to World Bank (half of them only from UK and Germany), which again appear to be more reflective of a homogenous sampling approach. Authors should also highlight the intrinsic limitations of quality coding in terms of reproducibility.
- On Page 8: “We undertook 38 semi-structured interviews”. Authors did not describe how sample size was determined. Authors should discuss how this small sample size might redound in uncertainty issues. The complete absence of statistical analyses of the data is striking. This affects the “empirical power” of the work, and one could question whether the described “trends” indicated in the manuscript are purely anecdotal, derived from sampling error and/or speculative.
-  “Like many kinds…” paragraph. This important paragraph highlighting the limitations of the study should be moved to the discussion/conclusion section.

D) The Results section in general:

 The reviewers wonder if the results section as a whole, which is relatively long in comparison to the rest of the paper, should be limited to Table 5 and its description and avoid the unending narrative of anecdotal specific responses. In case the authors feel the need to present all these quoted responses, perhaps they could be moved to the supplementary section.

- “Some participants…” “Many saw a preprint as…” “Other participants saw preprints..” “there were signs this…” and elsewhere. This vague and unclear wording could be avoided by the use of absolute numbers or percentages of total responses.
- Table 5: Please consider complementing the legend to provide definitions to “systemic” and “individual”.
- Table 6: Following Table 5 title, it would be more reasonable to entitle Table 6 as “ **potential** challenges of preprints”, or “possible” as depicted in the same table in Chiarelli *et al.* 2019.
- On Page 13: “One preprint service provider observed that **only** 10% of preprints received comments”. While this % is depicted as low in the text by the use of “only”, authors should contextualize this type of assertion. Peer reviewed articles published in journals are far less commented than preprints as reported elsewhere.
- On Page 14:  “author might invite comments from people who are expected to be positive about their work”. Again, this and other speculative statements should be leveled when they are employed to contrast with traditional peer-reviewed journal articles. It is a common practice in an important number of journals to ask authors to provide contact information of potential reviewers, which could eventually lead to the same issues expressed in the preceding statement.
- On Page 14: “We note…”. This relevant paragraph, highlighting the limitations and anecdotal nature of the study, should be moved to the discussion/conclusions.

E) The Discussion/Conclusion: As expressed above, some parts of the results section could be moved here as they reflect more speculative comments based on the interview responses.

 F) The References:

 This citation  “Sarabipour *et al*.: On the value of preprints: an early career researcher perspective. PeerJ Preprints (2018)” Is now peer-reviewed and hosted as “On the value of preprints: An early career researcher perspective. Sarabipour *et al. *PLoS biology (2019)[.

We confirm that we have read this submission and believe that we have an appropriate level of expertise to confirm that it is of an acceptable scientific standard, however we have significant reservations, as outlined above.

## References

[1] : Maintaining confidence in the reporting of scientific outputs.2018; 10.7287/peerj.preprints.27098v1 10.7287/peerj.preprints.27098v1

[2] : Preprints are good for science and good for the public. *Nature*.2018;560(7720) : 10.1038/d41586-018-06054-4 10.1038/d41586-018-06054-4 30158619

[3] : Preprints help journalism, not hinder it. *Nature*.2018;560(7720) : 10.1038/d41586-018-06055-3 10.1038/d41586-018-06055-3 30158616

[4] : Together scientists and journalists can spot poor preprints. *Nature*.2018;560(7720) : 10.1038/d41586-018-06053-5 10.1038/d41586-018-06053-5 30158617

[5] : Preprints: recall Nature’s nasty past. *Nature*.2019;570(7759) : 10.1038/d41586-019-01748-9 10.1038/d41586-019-01748-9 31164765

[6] : On the value of preprints: An early career researcher perspective. *PLoS Biol*.2019;17(2) : 10.1371/journal.pbio.3000151 e3000151 10.1371/journal.pbio.3000151 30789895PMC6400415

[7] : In praise of preprints. *International Journal of Systematic and Evolutionary Microbiology*.2019;69(7) : 10.1099/ijsem.0.003323 1841-1843 10.1099/ijsem.0.003323 30938668

[8] : Tracking the popularity and outcomes of all bioRxiv preprints. *bioRxiv*.2019; 10.1101/515643 10.1101/515643 PMC651053631017570

[9] : Releasing a preprint is associated with more attention and citations. *bioRxiv*.2019; 10.1101/699652 10.1101/699652 PMC691433531808742

[10] : The effect of bioRxiv preprints on citations and altmetrics. *bioRxiv*.2019; 10.1101/673665 10.1101/673665

[11] : Comparing quality of reporting between preprints and peer-reviewed articles in the biomedical literature. *bioRxiv*.2019; 10.1101/581892 10.1101/581892 PMC770620733292815

[12] : A proposal for the future of scientific publishing in the life sciences. *PLoS Biol*.2019;17(2) : 10.1371/journal.pbio.3000116 e3000116 10.1371/journal.pbio.3000116 30753179PMC6372143

# Response

**Published:** 2019-11-14

**Authors:** Andrea Chiarelli

**Affiliations:** Research Consulting, UK

**Summary:**

*In this manuscript Chiarelli et al. assess preprints from the point of view of a diverse group of stakeholders. The authors perform an analysis based on 38 interviews with representatives of research funders, research performing organizations, preprint servers and service providers; and researchers (engaged and unengaged). Additional discussion is provided on the benefits and challenges of preprint posting, along with issues such as infrastructure and financial sustainability and the definition of a ‘preprint’ in different communities, and the impact this has on further uptake. This study provides a thorough investigation on an emerging and relevant topic, giving a platform to the opinions of under-represented stakeholders on the discussion of preprints: a key driver of the transformation of the scholarly publishing landscape.*

**Authors’ response:**

 We are grateful for the comments made by the reviewers. They have been extremely useful for us in reviewing our approach. We have made changes in response to the suggestions of the reviewers wherever possible and have explained our responses in detail below.

***Reviewers’ comments:***

*We would like to ask the authors to address the following:*

**

*The introduction may benefit from additional literature to balance the many sections. For instance:*

**

*1. On Page 3: “However, skeptics have questioned the value of preprints and even suggested they may be dangerous – circulating versions of articles before they have been quality controlled by peer review may lead to unnecessary risk, particularly in disciplines like medicine (Sheldon, 2018).” The only negative note on preprints by Sheldon 2018 was well rebutted by at least 5 publications:*

- *Maintaining confidence in the reporting of scientific outputs - Sarabipour et al. (2018)* *^1^* *.*
- *Preprints are good for science and good for the public - Sarabipour (2018)* *^2^* *.*
- *Preprints help journalism, not hinder it - Tennant el al. (2018)* *^3^* *.*
- *Together scientists and journalists can spot poor preprints - Fraser & Polka (2018)* *^4^* *.*
- *Preprints: recall Nature’s nasty past - Cobb (2019)* *^5^* *.*

 

**Authors’ response:**

 We are happy to comment that this point has been rebutted. Prompted by this comment, we have added a paragraph in the literature review on the fact there has been vigorous debate on this and a number of other aspects of preprints. We believe it is worthwhile explicitly acknowledging this, as follows:

 “It is noticeable that the literature on the pros and cons of preprints has, like many aspects of open science, given rise to robust discussion and debate. The paper by Krumholz, Ross, & Otto (2018) cited above is itself structured as a debate, with the first two authors making the case for preprints and the third expressing concerns. Sheldon’s (2018) opinion piece in Nature arguing that preprints could have a negative impact beyond the scientific community, was met with vociferous rebuttals in the letters pages of the journal the following month (Fraser & Polka, 2018; Sarabipour, 2018; Tennant, Gatto, & Logan, 2018). On social media, such as Twitter, there have also been vigorous exchanges (e.g. Twitter, 2019).”

 For completeness, we have also added additional references to sources raising concerns about the adoption of preprints within medicine (Krumholz, Ross, & Otto, 2018 and Leopold et al, 2019). The article by Leopold et al is particularly significant in that it is not just a contribution to the debate but is the (re-)statement of an editorial policy for several medical journals who follow the Ingelfinger rule, clearly stating arguments against preprints. We believe this remains a live debate, and have sought to reflect this in the revised article.

 Apart from this particular point, a large number of comments made by the reviewers which we respond to in detail below relate to how we present the literature and the wider debate in our introduction and literature review. It is, therefore, worthwhile clarifying our approach in general terms.

 In our literature review, we report various views on preprints – their features, their perceived advantages and disadvantages etc. We do so in an evaluative way but aim to represent different perspectives. We agree with the reviewers, balance is important. We, therefore, spend as much time discussing perceived benefits as portrayed in the literature as we do challenges – and our account is constructed in a way specifically to create balance.  

 Our introduction and literature review sets the scene for our research – the concern about quality and potential misuse of results features in the literature as well as in our interview data, for example, and so discussing the first of these helps to contextualise the second. Our review of the literature is not an opinion or advocacy piece (although much of the literature we cite fit these categories). Our literature review is not designed to set out a particular position (for or against) in relation to preprints, nor would that be appropriate for a study of this kind. Rather, it aims to report the literature in a balanced way.

 Krumholz, H. M., Ross, J. S., & Otto, C. M. (2018). Will research preprints improve healthcare for patients? *BMJ*, *362*, k3628. https://doi.org/10.1136/BMJ.K3628

 Leopold, S. S., Haddad, F. S., Sandell, L. J., & Swiontkowski, M. (2019). Editorial: Clinical Orthopaedics and Related Research, The Bone & Joint Journal, The Journal of Orthopaedic Research, and The Journal of Bone and Joint Surgery will not accept clinical research manuscripts previously posted to preprint servers. *Clinical Orthopaedics and Related Research*, *477*(1), 1–4. https://doi.org/10.1097/CORR.0000000000000565

***Reviewers’ comments:***

*Articles 1-4 above address the relationship between scientific reporting and journalism. Further to the authors remark on “unnecessary risk particularly in disciplines like medicine” - preprint servers carry a highly visible note on preprinted manuscript stating that these research products are not peer-reviewed.*

**Authors’ response:**

 The point we are making here is that criticism of preprints has occurred in the literature based on the argument that they are potentially dangerous. Once again, we are reporting the debate in the literature, we are not writing an advocacy/opinion piece ourselves. We are not saying we agree or disagree with the arguments, rather we are reporting them to be part of the current debate.

 As noted above, we have added two further citations to our paper to further illustrate the point that this concern has been raised in the literature (Krumholz, Ross, & Otto, 2018 and Leopold et al, 2019). In the article by Krumholz et al (another opinion piece, this time in the form of a debate between supporters and sceptics of preprints), Otto argues, “Medical research preprints are unnecessary for scientific progress and dangerous for patient care”. Interestingly, the guidance on preprints produced by COPE ( https://publicationethics.org/files/u7140/COPE_Preprints_Mar18.pdf) acknowledges that such criticisms have been made of preprints (even if it is not an argument that it supports):

 “Some critics have raised concerns that preprints may have a negative impact on the credibility and public perception towards research, since the information has not been scrutinised and validated via peer review. For example, what are the risks if a preprint with a potential impact on public health is interpreted by some as established evidence?”

 The fact that preprints are labelled as such, as the reviewers point out, helps to mitigate the possible misuse of preprints, but does not eliminate that danger. In an open environment, there are possibilities that medical research could be accessed by patients, campaigners and others directly. There are well known examples of this happening and of the impact of misunderstandings being magnified by social media. Even if many of the concerns are hypothetical (as our research suggests), they cannot be simply dismissed, and need to be reported in any overview of the literature.

***Reviewers’ comments:***

*Regarding biomedical and medical preprints and risk to public: This is not a compelling argument since care is delivered to patients by physicians.* *MedRxiv* *was recently established and is accepting manuscripts to accelerate medical research. The server front page notes that: “Caution: Preprints are preliminary reports of work that have not been peer-reviewed. They should not be relied on to guide clinical practice or health-related behavior and should not be reported in news media as established information.”*

**Authors’ response:**

 Our point, again, is that the debate in the literature raises this concern. And it needs to be acknowledged, we believe, in any fair reporting of the current debate. We refer to our response above. We would add that open-access medical content can more easily be accessed directly by non-clinicians without expert mediation. In fact, the whole literature on the ‘expert patient’ builds on this as a positive. There is however the risk of misunderstanding. That is one point made by Otto (see above) and also by Leopold et al. Note MedRxiv had not been released when we carried out our data gathering, although one participant anticipated the kind of screening and flagging of preprints mentioned in the reviewers’ comments above.

***Reviewers’ comments:***

*2. On Page 3: “the current and potential future role of preprints as a vehicle for scholarly communication” - there are and will be many role(s) for preprints in the current and future of the scholarly endeavors and they are summarized here:*

- *On the value of preprints: An early career researcher perspective - Sarabipour et al.(2019)* *^6^*
- *https://asapbio.org/reading*
- *In praise of preprints - Fry et al. (2019)* *^7^* *.*

 

**Authors’ response:**

 Agreed. These papers make the case for preprints in the current scholarly communication system. We have cited Sabipour et al (2019). We will happily add reference to the more recent explanation of the advantages of preprints by Fry et al (2019). These papers (for the most part, at least) confine themselves to discussing the role of preprints in the current scholarly communication environment rather than discussing how they might contribute to more radical change in future. We have however added to our Discussion a paragraph with some more information in this area. This discusses the overlay idea amongst others:

 “The future of preprints servers and their links with the overall scholarly communication process and infrastructure remain unclear. It is possible that the recent rise in preprint services might be reversed and that preprints go through a period of retrenchment, returning to serve the core areas traditionally associated with preprint use, such as high-energy physics. A possible alternative to such a ‘retrenchment scenario’ is what might be called the ‘patchiness scenario’, where different levels of adoption exist across different fields. Patchiness may be an ongoing situation, or may be a transition stage towards a possible ‘ubiquity scenario’. For preprints to become ubiquitous, of course, requires significant cultural and infrastructural change, some of which is indicated by the data presented here. This may partly depend on the integration between preprint services and other parts of the scholarly communication infrastructure and on related cultural norms. Closer integration may give rise the possibility of more radical change in scholarly communication, creating opportunity for developments, such as overlay journals. Overlay journals have been discussed as thought experiments since at least the turn of the 21st Century (A. Smith, 2000; J. W. T. Smith, 1999) and there have been notable experiments in this area, and some ongoing services in specialised areas do exist, such as Discrete Analysis (Ball, 2015). However, we are yet to see their widespread adoption, even though the potential remains.”

***Reviewers’ comments:***

*3. On Page 4: “much of the literature is still to be found in editorials and opinion pieces rather than data-driven research” - the fact is that the number of data driven literature on preprints is growing rapidly and are noteworthy. Examples are:*

- *Tracking the popularity and outcomes of all bioRxiv preprints - Abdill & Blekhman (2019)* *^8^* *.*
- *Releasing a preprint is associated with more attention and citations - Fu & Hughey (2019)* *^9^* *.*
- *The effect of bioRxiv preprints on citations and altmetrics - Fraser et al. (2019)* *^10^* *.*
- *Comparing quality of reporting between preprints and peer-reviewed articles in the biomedical literature - Carneiro et al. (2019)* *^11^* *.*

**Authors’ response:**

 Agreed. All of these papers have been made available in 2019 in various forms (all of them since we gathered our data, two of them since we submitted our paper to *F1000*). They comprise a new empirical base for preprints studies. We have adjusted our literature review to take these recent additions to the literature into account, adding a new section on ‘The use and impact of preprints’:

 “The use and impact of preprints. A noticeable recent development in the literature has been publication of a number of empirical studies on the use and impact of preprints. These include Carneiro et al.’s (2019) study which compared the quality of reporting of findings in preprints from PubMed and bioRxiv against formally-published journal articles based on a number of criteria tested through a questionnaire...” etc.

***Reviewers’ comments:***

*4. On Page 4: “Accessibility (4) is crucial, with a preprint normally defined as being (or assumed to be) openly available: it “can be viewed without charge on the Web” and “Therefore, the venue for distribution of preprints is often assumed to be a freely-accessible server of some kind”. It is a fact that all preprint servers are freely accessible to any human being that has access to internet around the world: free of charge to submit by authors, free of charge to read by all anywhere on the planet.*

**Authors’ response:**

 Agreed. And that is why we have included open accessibility in our definition of what constitutes a preprint. When discussing preprints that is the way many authors of the literature define them, or at least that is a characteristic they assume. Sometimes this is taken for granted amongst commentators but is always at least implicit. We have made changes to our discussion of definitions to make this clearer. In Table 1, we have changed the description of this component to, “Preprints are openly available online”. In the accompanying textual discussion, it now reads as:

 “Accessibility (4) is crucial in definitions. A preprint is normally defined as being (or assumed to be) openly available online: it “can be viewed without charge on the Web” (Berg et al., 2016, p. 899). The idea of openness is fundamental to discussions on preprints. The venue for distribution of preprints is often assumed to be a freely-accessible server of some kind, a point highlighted by Berg et al. (2016, p. 899), who include in their definition that a preprint “is uploaded by the authors to a public server”.”

***Reviewers’ comments:***

*5. On Page 5: “To say that an output is “deserving” of dissemination is, of course, a value judgement and difficult to demonstrate for each deposit as it is made, but it is one that is implicit in much of the discourse on preprints.” - this is not entirely true. The value of preprints are well known/articulated/discussed at this point as preprints accelerate research and altmetric attention to scientific work: Please see references 8-11 above. Preprints have been deposited on arXiv for nearly three decades now and are invaluable to the physical sciences community. Other forms of pre-peer review material such as computer code and protocols have been disseminated open source and their value is well noted in various fields.*

**Authors’ response:**

 What we are discussing here is how preprints are defined in the literature. Bourne et al. (2017) extend the definition of a preprint beyond that of a version of a peer-reviewed paper before it is peer-reviewed and formally published to include “a research output that has not completed a typical publication pipeline but is of value to the community and deserving of being easily discovered and accessed”. What we are pointing out is that it is interesting that this definition defines a preprint as something that is “deserving” of dissemination. To state that something is “deserving” of dissemination is a value judgement. To incorporate a value judgement in a definition is unusual. Here we are not discussing whether preprints *are* valuable or not, rather we are discussing whether value should be part of the definition of what constitutes a preprint. Of course, particular preprints may or may not be valuable. Preprints as a whole may or may not be valuable. But to *define* preprints as what is valuable is somewhat curious. If an output has “not completed a typical publication pipeline” but is *not* deemed to be valuable, does that mean it is not a preprint? And if so, who decides what is a preprint or not, and on what basis? By extending the definition in this way Bourne et al have at least created an ambiguity.

***Reviewers’ comments:***   

*6. On Page 5: “dis-benefits of preprints” - unless the authors may provide any evidence of “dis-benefits of preprints” besides one note (Sheldon 2018), it is best to avoid using this word “dis-benefits” and consider rephrasing to “perceived dis-benefits”. Please see reference 1-5 above.*

**Authors’ response:**

 We have changes “dis-benefits” to “challenges”. We now refer to “Perceived benefits and challenges” of preprints. This is a fairer way to refer to the current debate, especially as most of the outputs we refer to discussing benefits and challenges are opinion pieces which present arguments – some more cogent than others – but only limited empirical evidence.

***Reviewers’ comments:***

*7. On Page 5: “Early dissemination can be useful to some particular members of the scholarly community, with early career researchers (ECRs)” - preprints and all other forms of scholarly pre-publication material are beneficial to ALL researchers not jut ECRs. Timely release of scientific results in the form of preprints and other forms such as code and data bases have already accelerated research by encouraging sharing of ideas, resources and as discussed in previously suggested references above.*

**Authors’ response:**

 Agreed. We have rephrased this to make (we hope) the point we are making clearer i.e. some of the literature emphasises the particular benefits to ECRs. This is not to say preprints are not useful for other groups, it is just that the benefits for this particular group are emphasised in some of the literature. The rewording is:

 “Early dissemination is seen by some as especially useful to a number of members of the scholarly community in particular, with early career researchers (ECRs) commonly identified as specific potential beneficiaries, as preprints can allow them to rapidly achieve “visibility” and demonstrate productivity in job and grant applications (Desjardins-Proulx et al., 2013; Sarabipour et al., 2018; Tennant et al., 2019).”

***Reviewers’ comments:***

*8. On Page 5: “Perhaps the most prominent criticism of preprints relates to this last issue: the lack of quality assurance through peer review (Sheldon, 2018). As well as a general concern about lowering quality standards, lack of quality control has been seen as potentially dangerous as “reports that have not undergone formal peer review [organised by a journal] could be misleading” (Lauer et al., 2015). Furthermore, uncertified science might be reported prematurely in the media and might even give rise to ‘fake news’ (Sheldon, 2018). Some insist that, at the very least, the opportunity to disseminate knowledge rapidly without peer review may encourage academics to produce low-quality outputs on fashionable topics (Teixeira da Silva, 2017).” The authors should declare that there are no evidences supporting all these bold statements. The Sheldon (2018) news article in fact explicitly states its speculative nature in the title which reads “preprints could promote confusion and distortion”. Please see references 1-5 above.*

**Authors’ response:**

 As stated above, we have added two other references to articles critiquing preprints to this paragraph – one of these (Otto) uses the term “dangerous” in relation to preprints. The other (Leopold et al) states directly, “We believe that the benefits proposed by advocates of medical preprint servers can be better achieved in other ways, and that medical preprint servers pose serious health and safety dangers to the patients for whom are supposed to be caring”. This is a view evident in the literature (as well as widely held amongst researchers and practitioners). We, therefore, need to report it.

 We refer to our comments above, that we are not writing an opinion piece here – rebutting arguments point by point we may disagree with; rather, we are reporting the contours of the debate in the literature. It is appropriate, we believe, to report this as an important argument which occurs in the literature.

***Reviewers’ comments:***

*9. On Page 5: “Others have gone further and questioned the value of self-appointed reviewers, as opposed to those selected by journal editors (see the issue of “self-policing” highlighted by Harnad, 1998).” This is a misleading claim. Voluntary reviews of preprints are done by researchers in that field or community and are not self-appointed by the authors. The open access nature of preprints allows the potential assessment of manuscripts by more than 2-3 (more than journal reviewers) normally and there is no evidence on these reviews having different quality than journal peer-reviews.*

**Authors’ responses:**

 The phrase “self-appointed reviewers” means reviewers who are self-appointed i.e. they appoint themselves, not that the authors appoint them. We hope this clarifies our point.

***Reviewers’ comments:***

*10. On Page 5: “Preprint posting, however, is unlikely in any case to substitute for the valuable role played by selective journals in filtering content”. This is an unfounded claim as a system of preprint servers and overlay journals may very well replace the outrageously expensive and time-consuming system of for profit journal publishing in under a decade. Please see reference 12.*

- *A proposal for the future of scientific publishing in the life sciences - Stern & O’Shea (2019)* *^12^* *.*

**Authors’ response:**

 Proposals of overlay journals have been around for at least 20 years (A. Smith, 2000; J. W. T. Smith, 1999). One of us wrote about such possibilities in 2004 (Pinfield, 2004); ideas further developed a decade ago (Pinfield, 2009). We agree, that the overlay model has real potential but are yet to see significant adoption of it (although interesting experiments and even some specialised services do exist). However, even in an overlay model filtering for quality is important and involves peer review. In the short/medium-term this function is normally seen as coming from journals since other peer-review providers are not commonly available.

 Note again, however, here we are still providing commentary on literature and are not advocating any particular approach ourselves. We have, however, changed the text of this sentence to make that clearer:

 “Preprint posting, however, is not normally seen as a substitute for peer review, currently managed by journals, in filtering content (Suber, 2012), a process that is commonly valued, even if recognised to be imperfect (Lee, et al., 2013).”

 Also, we have added a paragraph (as previously mentioned) in the Discussion on possible futures of preprints servers and their connections to the wider scholarly communication infrastructure, including overlay journals, since we agree, there is considerable potential in the overlay model.

 “The future of preprints servers and their links with the overall scholarly communication process and infrastructure remain unclear… Closer integration may give rise the possibility of more radical change in scholarly communication, creating opportunity for developments, such as overlay journals. Overlay journals have been discussed as thought experiments since at least the turn of the 21st Century (A. Smith, 2000; J. W. T. Smith, 1999) and there have been notable experiments in this area, and some ongoing services in specialised areas do exist, such as Discrete Analysis (Ball, 2015). However, we are yet to see their widespread adoption, even though the potential remains.”

 Pinfield, S. (2004). Self-archiving publications. In G. Gorman & F. Rowland (Eds.), *Scholarly Publishing in and Electronic Era: International Yearbook of Library and Information Management 2004-2005* (pp. 118–145). Retrieved from http://eprints.nottingham.ac.uk/142

 Pinfield, S. (2009). Journals and repositories: An evolving relationship? *Learned Publishing*, *22*(3), 165–175. https://doi.org/10.1087/2009302

 Smith, A. (2000). The journal as an overlay on preprint databases. *Learned Publishing*, *13*(1), 43–48. https://doi.org/10.1087/09531510050145542

 Smith, J. W. T. (1999). The deconstructed journal – a new model for academic publishing. *Learned Publishing*, *12*(2), 79–91.

***Reviewers’ comments:***

*11. On Page 5: “Whilst this convention has come under criticism and been withdrawn by some publishers, it still exists for some journals, e.g. in medicine and chemistry (Lauer et al., 2015; Teixeira da Silva & Dobránszki, 2019)” - this sentence is misleading. In the current scenario, authors should consider changing “some” to “most” publishers.*

**Authors’ response:**

 Amended.

***Reviewers’ comments:***

*12. On Page 6: “For example, it is not universally agreed when an output should be citable (in the literature, funding proposals or promotion cases)”. It is now becoming more and more agreed upon that preprints should be cited in articles and research grant proposals. Major funding bodies such as NIH, CZI and Wellcome Trust allow and encourage citation of preprints. Many journals allow citation of preprints and other research outputs (eLife and PLOS family of journals are examples).*

**Authors’ response:**

 We agree that it is becoming more common to accept citation of preprints in articles and grant proposals, and we have cited instances of this in our paper: “Perhaps the most noticeable shift recently in terms of policy is that of funder policies. Some funders have now explicitly signalled support for use of preprints, including allowing citation of preprints in funding bids, and support their inclusion in cases for academic advancement (Berg et al., 2016; Bourne et al., 2017).” However, our statement that this is not “universally” the case is still correct and is a measured way of making the point.

***Reviewers’ comments:***

*13. On Page 6 and other pages: “Scooping”, and “a claim of precedence”: Scooping is a perceived concern of researchers and is unfounded. Many researchers present unpublished research at conference. Perceived preprint concerns and claim of precedence have been discussed in reference 6 above.*

**Authors’ response:**

 Yes, we agree that many of the concerns expressed in the area of scooping are unfounded. However, the point is that concerns are expressed in this area, and that is what we are reporting. Like many of the other areas we report from the literature, this area is also raised in our dataset. This issue is addressed in the ASAPbio site, which also discusses the EMBO “scoop protection” initiative: “If an author submits a manuscript within 4 months of posting a preprint, EMBO will consider the work novel even if a competitor publishes similar work during that time.” The fact that such protections are put in place in this way demonstrates that the issue is still a concern for many. As an advocate of preprints, Berlin (2018) still states, “While some journals already guarantee a “scooping protection”, this is not common practice and most authors are still at risk of being “scooped””. What we are reporting here is the concern.

 Berlin, S. (2018). If the papers don’t come to the journal…. *EMBO Reports, 19*(4). https://doi.org/10.15252/embr.201845911

***Reviewers’ comments:***

*14. On Page 6: “or when it can be used to establish a claim of precedence” and “In disciplines where a preprint is not considered appropriate to establish precedence, it has also been suggested that making a preprint available may actually encourage research to be scooped by rival researchers who publish in a recognized journal before the preprint authors (the ‘flip side’ of the priority claim argument above) (Kaiser, 2017).” Regarding these repeated sections on scooping and priority claims:*

- *2-3 papers published via preprints or journals in close temporal proximity of each other have all been projects 2-4 years in making so it is very difficult to assess who did it first.*
*  *
- *Who gives researchers priority? A preprint that is posted online has a date and that gives precedence to the work. In reality, as researchers we all know that priority is given to researcher by their field and their peers. Scientific research is often presented at conferences well in advance of preprints or journal publication. As we know what matters in research is scientific reputation and that is given to researchers by their peers and field during a career in scientific research.*
*  *
- *Multiple papers published via preprints or journals in close proximity of each other only show the reproducibility of one another are need to be celebrated not feared so repeated arguments on scooping and priority claims do not move a positive conversation on research forward specially for early career researchers and the future generations of researchers.*

 

**Authors’ response:**

 Yes, these are useful and interesting points. It is not clear from them, however, how we might change our paper in response. The key point we are making in our literature review is that scooping is identified as a concern in the literature. It is also expressed as a concern within our interviews, demonstrating it is a live issue in this space. The fact that preprints are often argued to be a way of asserting priority is itself evidence that researchers fear being scooped – in that case using that fear as an argument to deposit preprints. Either way, the key point we are making in our literature review is that scooping is raised as an issue, and that is what we are reporting.

***Reviewers’ comments:***

*15. On Page 6: “Some have argued that initiatives such as Declaration on Research Assessment (DORA), with its emphasis on the quality of the output rather than venue of publication, promote use of preprints (Polka, 2018)”. It is noteworthy that DORA has now become a major international initiative and is signed by over 1,400 organizations including major universities and other research institutions and 14,000 researchers.*

**Authors’ response:**

 Yes, once again we agree. We expect that most readers of this paper will be familiar with DORA and as it is not the focus of our study, we do not discuss it in detail. If readers wish to know more, we have now provided a reference in our paper to online information about the initiative.

**Other comments to the MS text:**

***Reviewers’ comments:***

*A) The Title:  Authors could consider an alternative title “Preprints and Scholarly Communication: a preliminary/exploratory study on Adoption, Practices, Drivers and Barriers”*

**Authors’ response:**

 We have changed the title to, “Preprints and Scholarly Communication: An Exploratory Qualitative Study of Adoption, Practices, Drivers and Barriers”.

***Reviewers’ comments:***

*B) The Abstract:*

- *“Our study is the first using empirical data to understand” - please change to: Our study **uses **empirical data… (See comments below highlighting other empirical studies on the topic).*

**Authors’ response:**

 Noted and changes made as outlined above and below.

***Reviewers’ comments:***

- *“The main concerns are related to the lack of quality assurance and the ‘Ingelfinger rule’.” Please change to: main **perceived **concerns.*

**Authors’ response:**

 We have added “perceived” to our discussion of benefits and challenges of preprints (as mentioned above). Here we refer to “concerns”. Concerns, by definition, relate to people's perceptions of the challenges. “Concerns” is not being used here as a synonym of “disadvantages” or “challenges” but rather to represent perceived disadvantages. “Perceived concerns” is tautological, and so we have limited our use of “perceived” in relation to benefits and challenges.

***Reviewers’ comments:***

*C) The Introduction:*

- *On Page 3: “key actors”. It is puzzling how the authors determined which the key stakeholders are in the preprints ecosystem. Authors should discuss why they did not incorporate “scientific journal publishers” as a relevant actor for this work. It is interesting that they incorporated “publishers” as node in Figure 1 as a stakeholder group but failed to incorporate “representatives” during their interviews.*
*  *

**Authors’ response:**

 In our study, we did acknowledge the important role of academic publishers but did not engage them directly. Our reason for this was that the publishing community is already discussing preprints in a structured way, e.g. via the work of COPE. Furthermore, we engaged directly a representative of MDPI (to discuss preprints.org) and representatives of F1000Research (to discuss their approach to pre-publication sharing), who are running preprint services.

 We have added an explanation of this in our Methodology and there is a note in the new limitations statement on this as well.

***Reviewers’ comments:***

- *On Page 3: “(Carà et al., 2017) .” Please delete the space.*

**Authors’ response:**

 Corrected.

***Reviewers’ comments:***

- *On Page 3: “The study is the first using empirical data”.  Please see our comment on the abstract.*

**Authors’ response:**

 We have removed this sentence. We believe that our study is the first qualitative study on the new wave of preprints, nevertheless. As acknowledged above several important quantitative studies have appeared (most still in preprint form) in 2019.

***Reviewers’ comments:***

- *On Page 3: “Zuckerberg Foundation” - the correct name is the Chan-Zuckerberg Foundation (CZI).*

**Authors’ response:**

 Corrected.

***Reviewers’ comments:***

- * On Page 3: “The study is the first using empirical data”. Please see the comments on the abstract above.*

**Authors’ response:**

 Corrected.

***Reviewers’ comments:***

- *On Page 4: “…relatively small number of peer-reviewed studies focusing on preprints – much of the literature is still to be found in editorials and opinion pieces”. The authors surely know that in general opinion pieces published in scientific journals are also peer-reviewed.*

**Authors’ response:**

 We have changed this sentence to avoid ambiguity: “Nevertheless, much of the literature is still to be found in editorials and opinion pieces rather than data-driven research.”

***Reviewers’ comments:***

- *“Defining preprints. Key components. Table 1”. A relevant aspect of preprints that is not contemplated here is that preprints are published open access free of charge. Perhaps that important feature could be incorporated in component 4. Maybe this feature, highly relevant for unprivileged research communities, was neglected given the sampling strategy employed (see comment below).*

**Authors’ response:**

 This the core of key component 4. We have rephrased the description in Table 1 to make this clearer: “Preprints are openly available online” (as also described above).

***Reviewers’ comments:***

- *On Page 8: “The study adopted a **heterogeneous** purposive sampling approach, aiming to include a wide range of perspectives from actors in the area. Participants comprised **senior representatives**…”. If the authors selected senior representatives of each stakeholder categories, one could question how heterogeneous the sampling approach was. Moreover, authors mention the use of snowball sampling, which is linked to homogeneous sampling. In addition, participants correspond to only eight OECD High-income economies according to World Bank (half of them only from UK and Germany), which again appear to be more reflective of a homogenous sampling approach. Authors should also highlight the intrinsic limitations of quality coding in terms of reproducibility.*

 

**Authors’ response:**

 We respond to the specific point raised by the reviewers below, but before doing so we thought it would be useful to precede our responses with an explanation of our approach to qualitative research in general. We repeat this explanation from our response to the other set of peer review comments received and although lengthy it, we hope, helps to explain our approach. This is the case since some of the comments made by the reviewers in their report are as much about qualitative research in general as they are about our particular study, and so we would like to set out our approach in the round, as context to our detailed comments further below. The open peer review process may be helpful here, as the comments we make are openly available and help to explain our approach, even though not all of the detail can be incorporated in our final paper. However, we thought it would be useful to reproduce it here as it helps to contextualise our specific responses to issues raised by reviewers below.

 Our general approach in responding to the comments of the reviewers on qualitative methods is to try to achieve a balance between, on the one hand, providing clarity and further detail where necessary, and, on the other hand, avoiding over-extending an already long paper. So we have made some changes to the text of the paper with the aim of achieving greater clarity but provide more detailed explanations here in in this report in order to provide fuller explanations that might be too detailed for the paper itself.

 Our study uses well-established qualitative research methods. Bazeley (2013) states: "researchers engaging in a qualitative study focus on observing, describing, interpreting, and analysing the way that people experience, act on, or think about themselves and the world around them”. Qualitative research aims to collect and analyse “rich, deep data” (Bryman, 2015) which enable understandings of issues like motivations, beliefs, and values of participants in the contexts in which they act. It focuses on understanding the perspectives of participants, including where disagreement and conflict may exist. Qualitative research typically attempts not just to identify behaviours but also *meanings* associated with behaviours. It is often useful for encountering unexpected issues and influences. Qualitative methods are commonly deployed therefore in studying phenomena and contexts which are emerging, where issues need to be mapped out, and theory developed (which can then in turn potentially be tested in various ways, including quantitatively). We chose to explore the preprints space using these methods in order to gain a rich and deep understanding of the issues involved, specifically including varying motivations, differing behaviours, and conflicting perspectives.

 Qualitative data can take different forms and be gathered in different ways, but often take the form of text, e.g. transcripts of interviews, as in our study. In this case the transcripts *are the data* being analysed, and there are well-established methods for carrying out such analysis. Analysis is typically conducted inductively, identifying patterns which emerge from the data. This is normally done through a process like thematic analysis (used in this study) involving a number of steps, as explained by Braun & Clarke (2006), who are cited in our study. There are a large number of other texts that describe this process, albeit with variations e.g. Bazeley (2013), Bryman (2015), Maxwell (2013) etc. In the first stage of the process, the interviews are fully transcribed and then read and reread thoroughly by the researchers. Secondly, the entire dataset is coded – key features of the data being labelled at a detailed level. “Codes…serve as shorthand devices to *label, separate, compile* and *organize* data” (Charmaz, 1983). This is an iterative process involving constant comparisons across the dataset. It can be carried out in various ways, but in our case, we used the NVivo software which facilitates coding of large amounts of text. Coding is a kind of fracturing process, breaking down the data into small fragments, and so the third stage of thematic analysis involves assembling codes into groups or themes. Themes are identified iteratively through constant comparisons, often involved in mapping out themes in relation to one another in order to define and refine them. These were the processes we followed in this study.

 In reporting qualitative research, the themes are normally used as a framework, rather than the questions that were initially asked of participants. This is an important aspect of the reporting of an inductive study in order to represent the themes that emerged from the data rather than the initial framework of the questions asked. From analysis of these themes, theoretical insights should emerge. These could take the form of systematic explanations of a context; models of relationships, issues, challenges etc; sets of questions or hypotheses that need to be further investigated; etc. We have tried to provide some of these, albeit tentatively, in our study.

 Sampling in qualitative research is normally carried out in a purposive (or purposeful) way. This is described by Bryman (2015):

 “Purposive sampling is a non-probability form sampling. The researcher does not seek to sample research participants on a random basis. The goal of purposive sampling is to sample cases/participants in a strategic way, so that the sample are relevant to the research questions that are being posed. Very often, the researcher will want to sample in order to ensure that there is a good deal of variety in the resulting sample, so that sample members differ from each other in terms key characteristics relevant to the research question.”

 We describe our sampling approach as a “heterogeneous purposive sampling approach”, which we go on to define as, “aiming to include a wide range of perspectives from actors in the area”. The sample was heterogeneous in a number of respects: it contained representatives of different roles in the scholarly communication system, from different countries (and therefore policy environments), and with different views and levels of experience of using preprints. Participants were selected by us based on an analysis of the field, consultation with our funders and others, and then approached directly and invited to participate. We also advertised on email discussion lists and social media inviting participants, but only selected people to be interviewed who met our sampling criteria. One challenge was in finding researchers willing to be interviewed who were not engaged with preprints, and so in order to do this, we not only used the selection method of approaching people directly but also used “snowball sampling” – asking for recommendations of other people who we might approach from participants. As Bryman (2015) comments:

 “Purposive sampling often involves more than one of the approaches outlined… For example, it is quite common for snowball sampling to be preceded by another form of purposive sampling. In effect, the process entails sampling initial participants without using the snowball approach and then using these initial contacts to broaden out to a snowballing method.”

 This is what we did, resulting in a final sample of 38.

 Sample size is a controversial issue. There is no obvious right answer to the question of, “how many interviews are enough?” The essays addressing that question by well-known qualitative researchers edited by Baker & Edwards (2012) include a wide range of different views, depending on a number of factors. Bryman quotes Mason (2010) as having examined UK and Irish PhD theses using qualitative interviews, which identifies them as using a mean sample size of 31 and a median of 28. Bryman, in his chapter on reporting research, looks in detail at a sample journal article reporting semi-structured interviews which has a sample size of 20 (Jones, Leontowitsch, & Higgs, 2010). Our sample size of 38 is relatively large. One key point to emphasise, however, is that sample size in qualitative research is normally not determined in advance. Rather, the sampling approach will be decided and participants recruited within the sampling frame. The researchers will then make a number of judgements during the data gathering process about when sufficient data has been collected. Chief amongst these is the idea of “theoretical saturation”, where new data “no longer suggests new insights” (Bryman, 2015). This involves a number of judgements, involving a close engagement with the data in relation to the research objectives.

 Because qualitative research usually involves identifying areas of motivations, perspectives etc, there is commonly a need to allow participant anonymity. Often interviewees are less frank when they are ‘on the record’. Less frank interviewees tends to lead to a rather bland dataset which makes it difficult to understand the real contours of a landscape. In our study, we gained ethical approval to name participants and their organisations (with their permission) but not to associate any particular quotations with individuals. We have adopted a conventional way of dealing with this by identifying various groups amongst our sample which we do relate with each quotation (“Unengaged researcher”, “Preprint service provider” etc). Naming the group from which the interviewee being quoted came helps to contextualise each quotation, but does not compromise promised anonymity.

 Providing extensive extracts of participant quotations is a critical part of presenting findings from qualitative research. Since these quotations are illustrative of the themes identified, and are examples of the data collected, showing them in detail is an important way of demonstrating the validity of the inferences being drawn from the research.

 Baker, S. E., & Edwards, R. (2012). *How many qualitative interviews is enough?* Retrieved from http://eprints.ncrm.ac.uk/2273/4/how_many_interviews.pdf

 Bazeley, P. (2013). *Qualitative data analysis: Practical strategies*. London: Sage.

 Bryman, A. (2015). *Social research methods* (5th ed.). Oxford: Oxford University Press.

 Jones, I. R., Leontowitsch, M., & Higgs, P. (2010). The experience of retirement in second modernity. *Sociology*, *44*(1), 103–120. https://doi.org/10.1177/0038038509351610

 Maxwell, J. (2013). Designing a qualitative study. In L. Bickman & D. J. Rog (Eds.), *The SAGE handbook of applied social research methods* (pp. 214–253). https://doi.org/10.4135/9781483348858.n7

 To reply to the specific issue on sampling and its heterogeneity raised by the reviewers on heterogeneity and snowball sampling.

 Our sample was heterogeneous in various ways and we have explained this in text added to our methodology, as follows:

 “The study adopted a heterogeneous purposive sampling approach, aiming to include a wide range of perspectives from actors in the area, selected in a “strategic way” in order to address the objectives of the study (Bryman, 2015). The sample was heterogeneous in a number of respects: firstly, it contained representatives of different roles in the scholarly communication system; secondly, it included participants from different countries (and therefore policy environments); thirdly, it comprised interviewees and with different views and levels of experience of using preprints.”

 On snowball sampling; snowball sampling is not linked to either homogenous or heterogeneous sampling in itself. It depends how it is used. However, as we state above, it was used in conjunction with another sampling method, as a way of achieving heterogeneity in the sample, particularly to recruit researchers (and particularly unengaged researchers).

 Participants from eight different countries is a wide sampling frame for a study of this type. This study does not make any claims to cover issues to do with the Global South but does encompass a wide range of different policy environments present in the different countries covered.

 We have added more explanation of the limitations of the study in a new section in the methodology, explaining limitations, as previously mentioned.

***Reviewers’ comments:***

- *On Page 8: “We undertook 38 semi-structured interviews”. Authors did not describe how sample size was determined. Authors should discuss how this small sample size might redound in uncertainty issues. The complete absence of statistical analyses of the data is striking. This affects the “empirical power” of the work, and one could question whether the described “trends” indicated in the manuscript are purely anecdotal, derived from sampling error and/or speculative.*

**Authors’ response:**

 We have described issues of sample size in our general explanation above.

 The comments of the authors about the absence of statistical analysis from our reporting of findings needs comment. Ours is a qualitative study, using qualitative methods and qualitative analysis. It would be inappropriate to apply most quantitative approaches to our data. The “power” of qualitative findings of this sort (to use the term used by the reviewers) is in the richness of the data associated with issues of motivation, attitudes etc, not in any quantitative inferences drawn. We hope our detailed explanation of our approach to using qualitative methods is helpful in contributing to our response on this point. It includes the approach we took to inductively analysing themes in our data.

***Reviewers’ comments:***

- * ”Like many kinds…” paragraph. This important paragraph highlighting the limitations of the study should be moved to the discussion/conclusion section.*

**Authors’ response:**

 Conventions on this differ across journals, and to some extent across disciplines. We have noticed statements of this sort in the method sections of other articles in F1000, and so would prefer to keep this section (in its newly amended form) in the Methods section, since it relates directly to the methods just described.

***Reviewers’ comments:***

*D) The Results section in general:*

**

*The reviewers wonder if the results section as a whole, which is relatively long in comparison to the rest of the paper, should be limited to Table 5 and its description and avoid the unending narrative of anecdotal specific responses. In case the authors feel the need to present all these quoted responses, perhaps they could be moved to the supplementary section.*

**Authors’ response:**

 The specific responses of our participants are the *data* for this study. Presenting them in this way is conventional for this sort of research and doing so in detail demonstrates the validity of the inferences we are drawing. The specificity of the quotations is in the nature of qualitative research – that is where the richness of the data comes from.

 It is noticeable that in his book on social sciences research, Bryman presents a sample article in order to illustrate presentation of qualitative research (Jones, et al., 2010)). This sample article follows a similar pattern of presentation of thematic findings with commentary by the authors and quotations from participant interviews. Bryman comments, “it is striking that, in presenting their findings, Jones et al. (2010) use verbatim quotations to reinforce the point they are making… They do so by including the quotations as they go along to reinforce or illustrate points they are making about the themes they extracted from that data. This is quite a common approach to the use of verbatim interview quotations.”

 Jones, I. R., Leontowitsch, M., & Higgs, P. 2010. The experience of retirement in second modernity. *Sociology, 44*(1), 103–120. https://doi.org/10.1177/0038038509351610

***Reviewers’ comments:***

- *“Some participants…” “Many saw a preprint as…” “Other participants saw preprints..” “there were signs this…” and elsewhere. This vague and unclear wording could be avoided by the use of absolute numbers or percentages of total responses.*

**Authors’ response:**

 We have addressed this comment by amending wording to be as precise as possible within the constraints of a qualitative study. Once again, there is a balance to struck here. On the one hand, as a general principle, it is essential to recognise that qualitative research should not be analysed quantitatively. Inferences cannot be drawn from the data in any statistically valid way from quantitative analysis of a dataset like this, and it is important not to give the impression that they can. Certainly, including percentages would not be appropriate. In any case, the data is not sufficiently structured to allow for reliable identification of a type of response which could be reported quantitatively. Every datum in a qualitative dataset is actually unique and although it is possible to identify commonalties, it is not normally possible to say that a certain proportion of participants said one thing or another. This likely to lead inappropriate homogenisation of views.

 Furthermore, it is important not to confuse prevalence with significance. In a qualitative dataset, some ideas may be mentioned by a large number of participants, but this does not necessarily mean they are significant. Significance may not, of course, be easy to judge (and it is a matter of judgement based on careful analysis) but it is not a simple issue of numbers of times an issue is mentioned in the dataset. We have however made some attempts to identify prevalence of issues raised in discussing pros and cons of preprints, something which is possible (simply identifying topics mentioned), with the relevant caveats.

 On the other hand, it is perfectly reasonable to mention some quantitative terms, “the majority”, or “a minority” or if an issue was only mentioned by one or was mentioned by all participants. That is what we have tried to do within the bounds of what is defensible. 

***Reviewers’ comments:***

- *Table 5: Please consider complementing the legend to provide definitions to “systemic” and “individual”.*

**Authors’ response:**

 We have added definitions as follows: ““Systemic” significance relates to those factors with system-wide impact e.g. the broad scholarly communication system or disciplinary community; “individual” relates to those factors primarily affecting individuals or small groups.”

***Reviewers’ comments:***

- *Table 6: Following Table 5 title, it would be more reasonable to entitle Table 6 as “ **potential **challenges of preprints”, or “possible” as depicted in the same table in Chiarelli et al. 2019.*

**Authors’ response:**

 Agreed and done.

***Reviewers’ comments:***

- *On Page 13: “One preprint service provider observed that **only** 10% of preprints received comments”. While this % is depicted as low in the text by the use of “only”, authors should contextualize this type of assertion. Peer reviewed articles published in journals are far less commented than preprints as reported elsewhere.*
*  *

**Authors’ response:**

 The preprint service provider making the statement was making the point that it was relatively low. Interestingly, it is, however, comparable to levels identified in a recent study of PLOS journals, co-authored by one of us:

 Wakeling, S., Willett, P., Creaser, C., Fry, J., Pinfield, S., Spezi, V., … Medina Perea, I. (2019). ‘No comment’? A study of commenting on PLOS articles. Journal of Information Science, (Online first). https://doi.org/10.1177/0165551518819965

***Reviewers’ comments:***

- *On Page 14:  “author might invite comments from people who are expected to be positive about their work”. Again, this and other speculative statements should be leveled when they are employed to contrast with traditional peer-reviewed journal articles. It is a common practice in an important number of journals to ask authors to provide contact information of potential reviewers, which could eventually lead to the same issues expressed in the preceding statement.*

**Authors’ response:**

 Here we are reporting the views of participants. We have added a comment in our discussion about there being evidence of misunderstandings amongst some researchers. However, stepping in to engage with participants during the reporting of findings in this one particular instance would not be consistent with our approach or appropriate for this kind of research.

***Reviewers’ comments:***

- *On Page 14: “We note…”. This relevant paragraph, highlighting the limitations and anecdotal nature of the study, should be moved to the discussion/conclusions.*

**Authors’ response:**

 We have introduced a new section spelling out the limitations of our research. We again note that this is a qualitative piece of research, based on interviews, with text as the *data* being analysed.

***Reviewers’ comments:***

*E) The Discussion/Conclusion: As expressed above, some parts of the results section could be moved here as they reflect more speculative comments based on the interview responses.*

**Authors’ response:**

 We have moved a whole subsection of the results into the Discussion section. This is the section which models different preprint services based on ownership and contribution.

 “At a systemic level, the issue of sustainability emerged as critical from our work. Four models for delivering preprint services emerged from the data… This is a fundamental shift which has major implications for the way the role of preprints is understood and the way preprints services are configured.”

 This discussion is derived from our data, but does not directly report data, but rather systematises what is expressed by our participants. It is therefore appropriately located in the discussion.

***Reviewers’ comments:***

*F) The References:*

**

*This citation  ”Sarabipour et al.: On the value of preprints: an early career researcher perspective. PeerJ Preprints (2018)” Is now peer-reviewed and hosted as “On the value of preprints: An early career researcher perspective. Sarabipour et al. PLoS biology (2019)[.*

**Authors’ response:**

 Updated.
